# When and why direct transmission models can be used for environmentally persistent pathogens

**DOI:** 10.1371/journal.pcbi.1009652

**Published:** 2021-12-01

**Authors:** Lee Benson, Ross S. Davidson, Darren M. Green, Andrew Hoyle, Mike R. Hutchings, Glenn Marion

**Affiliations:** 1 Computing Science and Mathematics, University of Stirling, Stirling, United Kingdom; 2 Disease Systems, Animal And Veterinary Sciences, Scotland’s Rural College, Edinburgh, United Kingdom; 3 Institute of Aquaculture, University of Stirling, Stirling, United Kingdom; 4 Biomathematics and Statistics Scotland, Edinburgh, United Kingdom; Fundação Getúlio Vargas: Fundacao Getulio Vargas, BRAZIL

## Abstract

Variants of the susceptible-infected-removed (SIR) model of Kermack & McKendrick (1927) enjoy wide application in epidemiology, offering simple yet powerful inferential and predictive tools in the study of diverse infectious diseases across human, animal and plant populations. Direct transmission models (DTM) are a subset of these that treat the processes of disease transmission as comprising a series of discrete instantaneous events. Infections transmitted indirectly by persistent environmental pathogens, however, are examples where a DTM description might fail and are perhaps better described by models that comprise explicit environmental transmission routes, so-called environmental transmission models (ETM). In this paper we discuss the stochastic susceptible-exposed-infected-removed (SEIR) DTM and susceptible-exposed-infected-removed-pathogen (SEIR-P) ETM and we show that the former is the timescale separation limit of the latter, with ETM host-disease dynamics increasingly resembling those of a DTM when the pathogen’s characteristic timescale is shortened, relative to that of the host population. Using graphical posterior predictive checks (GPPC), we investigate the validity of the SEIR model when fitted to simulated SEIR-P host infection and removal times. Such analyses demonstrate how, in many cases, the SEIR model is robust to departure from direct transmission. Finally, we present a case study of white spot disease (WSD) in penaeid shrimp with rates of environmental transmission and pathogen decay (SEIR-P model parameters) estimated using published results of experiments. Using SEIR and SEIR-P simulations of a hypothetical WSD outbreak management scenario, we demonstrate how relative shortening of the pathogen timescale comes about in practice. With atttempts to remove diseased shrimp from the population every 24h, we see SEIR and SEIR-P model outputs closely conincide. However, when removals are 6-hourly, the two models’ mean outputs diverge, with distinct predictions of outbreak size and duration.

## Author summary

Mathematical models of the spread and progression of communicable disease in populations are important tools in efforts to prevent and control outbreaks. A common class of disease models assume that infection is transmitted directly from infectious to susceptible individuals when they are in close proximity—so called direct transmission models. These are used widely and have proven invaluable as simplified descriptions of a wide array of infectious diseases in diverse populations. However, many pathogens spread through indirect, environmental routes of transmission, for example via contact with contaminated water sources in the case of cholera, or inhalation of infectious airborne droplets for respiratory infections, such as Covid-19.

We show that direct transmission models work well for such pathogens with short environmental lifetimes and where hosts shed pathogens into the environment at high rates. This means that we do not require information about environmental pathogen levels to understand the behaviour of outbreaks caused by these pathogens. When shedding rates are also low, e.g., with macroparasitic infections, or when variable environmental factors play a role in transmissibility, then explicit modelling of both the pathogen and environmental transmission will provide a more accurate picture than a direct transmission approximation.

## Introduction

The famed Susceptible-Infectious-Recovered (SIR) compartmental model framework of Kermack and McKendrick [1], and its many subsequent extensions (see [2], for example), stand as prominent examples of what can be gained from simple models of complex systems. In addition to the assumption that the host population can be divided into a finite number of discrete states, transmission of infection within such models is characterised by a force of infection that depends linearly upon the numbers of infectious individuals. Throughout this paper, we call such a model a direct transmission model (DTM) (as in [3]). The proportionality constant, known as the transmission rate, is often interpreted as the rate at which individuals within the population come into contact with each other, times the probability that such a contact leads to the transmission of infection (termed an infectious contact in [4]), times the probability of successful transmission. However, this simplified representation has been extended, for example, to account for non-uniform frequency of contact among the population and levels of infectiousness varying across individuals and over time.

Such approaches have been pivotal in gaining valuable insights into the dynamics and patterns of the spread of disease throughout many varied populations. Recently, for example, DTMs have served as the basis for understanding drivers of spatial spread of Ebola virus [5], the likely effectiveness of scaling up certain vaccination, treatment and testing regimes in the fight to control hepatitis B [6] and the importance of targeting household transmission of MRSA as a preventative strategy [7]. Incorporation of a spatial element into the DTM framework enables the observed spatial-temporal trajectory of the 2001 foot and mouth outbreak in the UK to be closely replicated and provides insight for control [8, 9]. DTMs have also been recently drafted into the effort to understand and predict the dynamics of SARS-CoV-2 [10, 11].

Despite these sucesses, DTMs may not always be appropriate, e.g., when members of the host population are in contact with environmental sources of infection, such as pools of pathogens residing on surfaces or in water bodies. The focus of this paper is to critique and support the use of DTMs in describing the spread of disease in the presence of such environmental pools of persistent pathogens. Examples of relevant disease systems include cholera [12, 13], avian influenza [14, 15] and even respiratory infections, such as SARS-CoV-2 [16]. Our case study (in Case study: White spot disease in penaeid shrimp) focusses on infectious disease spread in aquaculture systems which are likely to feature a large degree of environmental transmission. In scenarios such as these it is prolonged exposure to these sources, in addition to direct infectious contact between individuals, that gives rise to new infections, in the most general case. Environmental transmission models (ETM, introduced below) are a second family of candidate models for such infections. As well as describing direct transmission of disease between hosts, ETMs also allow indirect, environmental routes of transmission due to interaction between the hosts and external pathogen, whose dynamics are in turn described in terms of shedding from infectious hosts as well as pathogen decay (or loss of viability). Epidemiological models, including DTMs and ETMs, are approximations of complex biological processes driving disease transmission and progression. The main goal of this paper is to show that the relative timescale separation between the host and pathogen populations determines whether environmental transmission due to contact with external pathogens shed by infectious hosts can be more parsimoniously described as direct transmission within the DTM framework. A key advantage of this approach is that we do not need information about environmental pathogen levels in order to fit DTMs.

A critical step in applications is the fitting of DTMs to data on disease outbreaks. Using Bayesian model fitting methods and graphical posterior predictive checks (GPPCs) that target observable characteristics of an outbreak, such as its final size and when it peaks, we show that DTMs fit very well to simulated host-disease event times that would occur when the infection is transmitted environmentally but the rates of pathogen emission and removal are high (Results). We show that this is explained by the force of infection within an ETM behaving increasingly like that of a DTM when the timescale of the pathogen population is shorter than that of the host population (Results). When fitted to simulated outbreak data, we find that DTMs still make accurate predictions of outbreak size and duration even when the underlying data generating process comprises a low rate of pathogen emission and long pathogen lifetimes. It is only the rate at which outbreaks grow towards their peak that is poorly predicted in such cases.

These issues are further highlighted in a case study illustrating the use of DTMs as approximate descriptions of outbreaks of disease due to an environmentally persistent pathogen (Case study: White spot disease in penaeid shrimp). Using parameter values estimated from published data on WSD infection in penaeid shrimp, we explore how imperfect interventions that aim to remove dead and diseased hosts at regular intervals impact outbreak control in closed populations of this aquaculture disease system. With removal attempts spaced at 24 hourly intervals, average outbreak trajectories, final outbreak size, outbreak duration, are accurately captured using a DTM, without need to model the pathogen load. When the frequency of the removal events are increased to every 6 h, we begin to see divergence between the two models, so that, e.g., the DTM predicts slightly larger outbreaks of shorter duration than the ETM. This case study illustrates the potential practical consequences of ignoring issues of timescale separation when applying DTMs to environmentally transmitted pathogens. Control and other processes in such disease systems may be accurately captured by DTMs at one timescale but are poorly represented at others; in this case underestimating the benefit of high frequency removal.

Tables 1 and 2 contain summaries of symbols and abbreviations used throughout this paper.

**Table 1:** Summary of symbols and their units used throughout this paper.

Symbol	description	units
*S*_*t*_, *E*_*t*_, *I*_*t*_, *R*_*t*_	numbers of susc., exp’d, infectious and rem’d hosts at time *t*	hosts
*N*	total host population size	hosts
*P*_*t*_	size of environmental pathogen pool at time *t*	virions
*α*	environmental transmission rate	virion^−1^ h^−1^
*β*	direct transmission rate	host^−1^ h^−1^
*ν*_*δ*_, *ν*_*γ*_	(gamma-distributed) exposed and infectious lifetime shape parameters	-
λ_*δ*_, λ_*γ*_	exposed and infectious lifetime rate parameters	h^−1^
*δ*, *γ*	(exponentially distributed) exposed and infectious lifetime rate parameters	h^−1^
*ϵ*	pathogen emission rate	host^−1^ h^−1^
*ρ*	pathogen decay rate	h^−1^
*f*(*t*)	force of infection at time *t*	h^−1^
**t**^*E*^, **t**^*I*^, **t**^*R*^	times of exposure, onsets of infectivity, removals	h
*m*	final outbreak size	hosts
*ω*_*β*_, *ω*_*γ*_, *ω*_*δ*_	prior exponential rate parameters for *β*, *γ*, *δ*	h^−1^
*κ*	host index corresponding to index exposure	-
$t_{\kappa }^{E}$	time of index exposure	h
*t*_peak_	time of outbreak peak	h
*I*_max_	size of outbreak at peak	hosts
*c*	relates to interpolated value for *t*_peak_	-
$\hat{\beta }\left(=\beta +\alpha \frac{\epsilon }{\rho }\right)$	effective direct transmission rate	host^−1^ h^−1^
$\hat{\delta },\hat{\gamma }$	reciprocals of mean simulated exposed and infectious lifetimes	h^−1^
$R_{0},{\hat{R}}_{0}$	basic reproduction ratio for SEIR / SEIR-P models	-
$\beta_{\text{ingest}},{\tilde{\beta }}_{\text{ingest}}$	rates of transmission due to ingestion in resp. Tuyen, et al. and Lotz & Soto	host^−1^ h^−1^
$\beta_{\text{cohab}},{\tilde{\beta }}_{\text{cohab}}$	similar to above for cohabitation	host^−1^ h^−1^
*α*_L_	lower estimate of *α* (Case Study)	ml virion^−1^ h^−1^
*ρ*_U_	upper estimate of *ρ* (Case Study)	h^−1^
*π*_E_, *π*_I_	probability of removal of exposed, infectious hosts (Case Study)	-

**Table 2:** Summary of abbreviations employed in the text.

Abbreviation	meaning
DTM	direct transmission model
ETM	environmental transmission model
DT (ET)	direct (environmental) transmission
SIR	susceptible-infectious-removed DTM
SEIR	susceptible-exposed-infectious-removed DTM
SIR-P	susecptible-exposed-infectious-removed-pathogen ETM
SEIR-P	similar to SIR-P
MCMC	Markov chain Monte Carlo
GPPC	graphical posterior-predictive check
DTA	direct transmission approximation
IDP	immigration-death process
SIWR	susceptible-infectious-waterborne reservoir-removed
WSD	white spot disease
WSSV	white spot syndrome virus

### Models for direct and environmental transmission of disease

#### A direct transmission model: Susceptible-Exposed-Infectious-Removed (SEIR)

**Fig 1:**
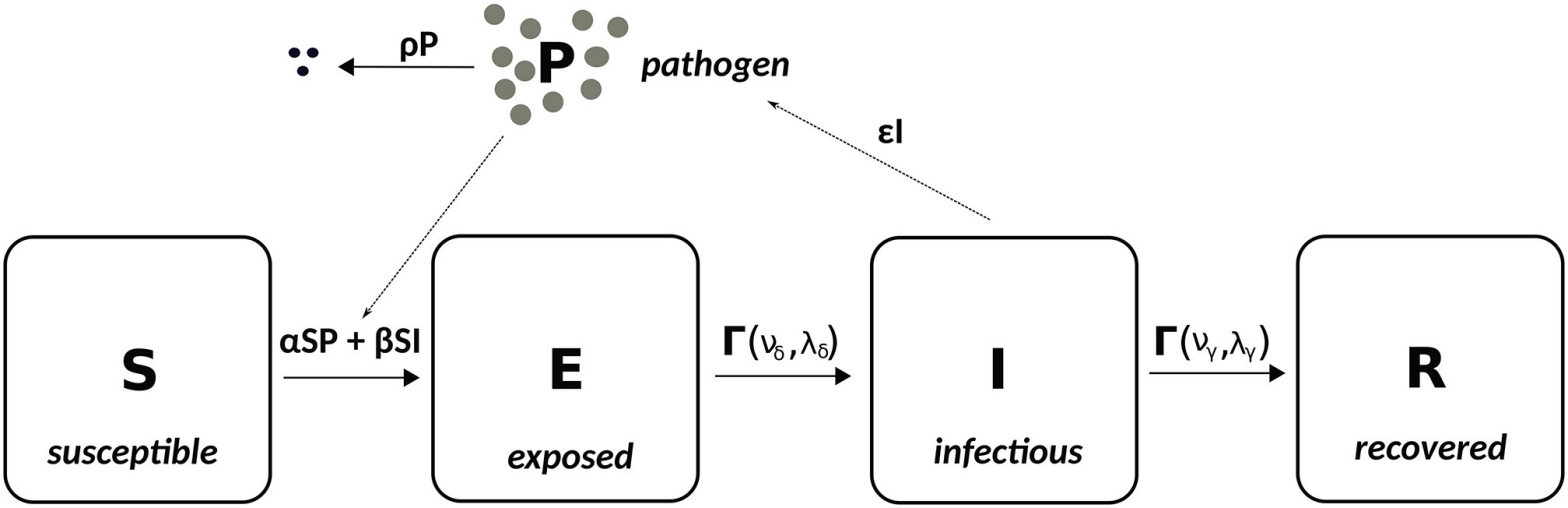
Susceptible-exposed-infectious-removed-pathogen (SEIR-P) environmental transmission model diagram illustrating the four host compartments ((S)usceptible, (E)xposed, (I)nfectious and (R)emoved, or recovered) and single pathogen compartment (P) of the model. The solid arrows indicate the movement of hosts between host compartments and the loss of viable pathogen from the system. The dotted arrows indicate how the host and pathogen parts of the model influence each other. The parameters *α* and *β* are the *environmental* and *direct transmission rates* while *ϵ* and *ρ* are the rates of pathogen emission and decay. The time that hosts spend in the E and I compartments is determined by the chosen *exposed* and *infectious* lifetime distributions, e.g., gamma distributions with shape parameters *ν*_*δ*_ and *ν*_*γ*_ and rate parameters λ_*δ*_ and λ_*γ*_, as shown here. When exponential distributions are assumed, then the rate parameters are denoted respectively by *δ* and *γ* (see Table 1 for a summary of all symbols used throughout the paper). When *α* = *ϵ* = *ρ* = 0 we obtain the SEIR direct transmission model as a submodel.

The stochastic, compartmental SEIR model (see Fig 1) referred to throughout this paper is a DTM that treats the focal population (the *hosts*) as divided into four sub-populations: hosts that are susceptible to disease (S), have been exposed to the disease but not yet infectious (E), infectious (I) and recovered or removed from the population (R). Hosts in the R compartment play no further role in the spread of disease. Here we consider outbreaks started by the introduction of a single host to a wholly susceptible population of size *N* (this is known as the *index exposure*), however, our results are generalisable to greater numbers of initial exposures. In addition a closed population is assumed, so that there is no immigration, births or non disease-induced mortality. Hosts remain in the E and I compartments for periods of time determined by the *exposed* and *infectious* *lifetime distributions*, i.e., random variables with continuous, positive distributions. In the case of exponentially-distributed lifetime distributions, with rates *δ* and *γ* respectively for the exposed and infectious states, the resulting process is a continuous time Markov chain and the current state of the system is fully determined by the number of hosts in the four compartments. The SEIR model may be specialised by stipulating that hosts spend a period of zero duration in the E compartment, resulting in a SIR model.

In Case study: White spot disease in penaeid shrimp we modify this model by additionally allowing, at regular intervals, each host in the E and the I compartments to go directly to the R compartment with probabilities *π*_E_ and *π*_I_. This represents regular attempts, with error, to remove all exposed and infectious hosts from the system.

The direct transmission assumption means that secondary cases are generated at a rate dependent on the number of infected individuals. Here we adopt the standard approach to modelling this. The probability that each susceptible host at time *t* becomes exposed to disease over the short time interval (*t*, *t* + *h*], is *βI*_*t*_*h* to first order, where *β* is the *direct transmission rate* and *I*_*t*_ is the number of infectious hosts present at time *t*. The force of infection, i.e., the rate of secondary infections, per susceptible host, or the imminent risk of infection that is faced by each susceptible host, at time *t* is therefore expressed as

$$\begin{array}{c} f\left(t\right)=\beta I_{t}. \end{array}$$ (1)

This means that the force of infection is proportional (by the factor *β*) to the number of infected hosts currently present. When the number of infected hosts increases or decreases, there is a corresponding instantaneous change in the force of infection. This is a key feature of this and other DTMs and represents an important modelling assumption.

#### An environmental transmission model: Susceptible-Exposed-Infectious-Removed-Pathogen (SEIR-P)

Here we define a class of models that represent both direct (DT) and environmental transmission (ET) of disease (see, for example, [17]) where the latter occurs via interaction of susceptible hosts with environmental pools of infectious pathogen. SEIR-P describes the time evolution of two populations: the hosts, divided into S, E, I and R sub-populations, measured in hosts (as in the DTM SEIR, described above) and the pathogen population (P—measured here in virions) in the environment, external to the hosts. When hosts enter state I they begin to emit pathogen at the fixed rate *ϵ*, i.e., they begin to contribute to an increase in the environmental pathogen load. The pathogen population decays exponentially at rate *ρ*.

Each susceptible host at time *t* now becomes exposed to disease over the short time interval (*t*, *t* + *h*] with probability (*αP*_*t*_ + *βI*_*t*_)*h* to first order, where the summand *βI*_*t*_ represents that part of the force of infection contributed by direct transmission, as in the SEIR model, and *α* and *P*_*t*_ represent the *indirect* or *environmental transmission rate* and size of the pathogen population (in virions) at time *t*, respectively. The force of infection is now

$$\begin{array}{c} f\left(t\right)=\alpha P_{t}+\beta I_{t}, \end{array}$$ (2)

which depends linearly on both the size of the environmental pathogen load and the number of infectious hosts. A change in the number of infected hosts now produces a delayed response in the force of infection due to the pathogen load taking time to either build up or decay.

Setting *α* to zero and restricting attention to the four host population compartments recovers the SEIR model described above and setting *β* = 0 produces a pure ET model. Finally, stipulating that hosts pass instantaneously from the E to the I compartment yields a SIR-P model with both DT and ET.

### The direct transmission approximation as timescale limit of SEIR-P process

**Fig 2:**
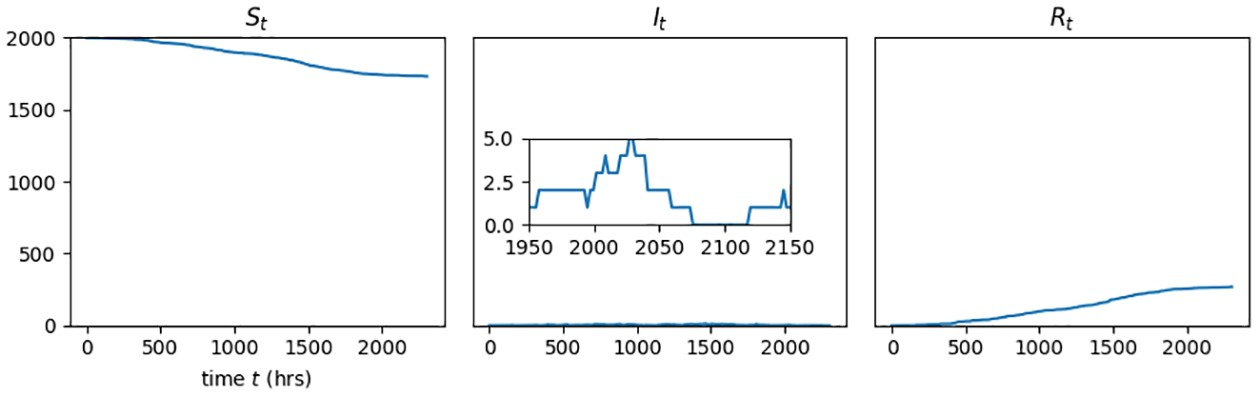
Host S, I and R outputs from a single realisation of SIR-P model with environmental transmission only (i.e., *β* = 0 host^−1^ h^−1^) among a host population of size *N* = 2000. The rates of environmental transmission and pathogen emission are *α* = 1.325 × 10^−10^ virion^−1^ h^−1^ and *ϵ* = 3462.5 virion host^−1^ h^−1^ and the rates of pathogen decay, *ρ*, and host recovery, *γ*, are both 0.029 h^−1^ (equivalent to a half-life of 24 h). The inset in the central panel is the key feature of interest and shows how the number of infectious hosts goes to zero roughly between the times *t* = 2075 h and 2120 h. Under the SIR model, as soon as the number of infectious hosts reaches zero no further secondary infections are possible. However, within the SIR-P model with comparable pathogen and host infectious lifetimes, outbreaks can appear to die off but later continue, due to the force of infection from long-living pathogens.

The two populations described by an ETM, the hosts and environmental pathogen, each have a timescale characterising their evolution, and the extent to which these differ has a qualtitative effect upon the behaviour of the model. For example, ETMs with shed pathogen retaining infectivity for durations comparable with the typical host infectious lifetime can exhibit outbreaks that appear to have died out, in terms of infected individuals, only to restart (see Fig 2). Similar behaviour is prohibited under the SIR model, where zero infectious hosts implies that there is no more force of infection to drive the outbreak forward. On the other hand, ETMs with short-lived pathogens produce host-disease dynamics that are reproducible with a host-only DTM, as we now demonstrate.

The probability that a susceptible host at time *t* becomes exposed within the short interval of time (*t*, *t* + *h*] is (*αP*_*t*_ + *βI*_*t*_)*h*, to first order. The relationship between the qualitative behaviour of the SEIR-P model and pathogen timescale comes down to the appearance of *P*_*t*_ in this expression. How does *P*_*t*_ behave? At time *t*, each infectious host is emitting pathogen at the rate *ϵ* so, overall, new pathogen is entering the population at the rate *ϵI*_*t*_. At the same time, the pathogen decays exponentially at rate *ρ*, or equivalently, each pathogen element remains viable on average for a duration 1/*ρ* (in appropriate units). This amounts to *P*_*t*_ being an immigration-death process (IDP) with inhomogenous immigration rate *ϵI*_*t*_ and death rate *ρ*. As a consequence of elementary theory of Markov chains (see, e.g., [18]), if we momentarily regard the number of infectives as fixed, *I*_*t*_ = *i* for *t* ≥ 0, then the distribution of *P*_*t*_ tends to $\text{Poisson}(\frac{\epsilon i}{\rho })$ and

$$\begin{array}{c} E\left(P_{t}\right)\to \frac{\epsilon i}{\rho }\;\text{as}\;t\to \infty . \end{array}$$ (3)

Increasing the values of the parameters *ϵ* and *ρ*, while keeping their ratio constant, increases the rate of convergence but has no other effect upon this limiting behaviour, with the limiting distribution unchanged. Returning now to variable *I*_*t*_, in the limiting case, *P*_*t*_’s behaviour can be characterised approximately as being in equilibrium, i.e., $P_{t}∼\text{Poisson}\left(\frac{\epsilon i}{\rho }\right)$ during intervals of constant *I*_*t*_ = *i*, and jumping without transition to a new equilibrium when *I*_*t*_ changes.

Consequently, for large *ϵ* and *ρ*, we may approximate the probability that a susceptible host becomes exposed over the interval (*t*, *t* + *h*] as

$$\begin{array}{c} \left(\alpha E\left(P_{t}\right)+\beta I_{t}\right)h=\left(\alpha \frac{\epsilon }{\rho }+\beta \right)I_{t}h=\hat{\beta }I_{t}h. \end{array}$$ (4)

The part of the SEIR-P model that describes the host disease dynamics is approximately a direct transmission SEIR model, with an *effective* transmission rate $\hat{\beta }$ and E and I lifetime distributions unchanged. This SEIR model is the *direct transmission approximation* (DTA) of the ETM.

This approximation of the sub-process, *P*_*t*_, is the stochastic analogue of the “quasi-steady state approximation” of the pathogen concentration discussed by Tien and Earn in their susceptible-infected-waterborne reservoir-removed (SIWR) ordinary differential equation model of cholera outbreaks among humans [19], in which the pathogen concentration in water sources is restricted to the “critical manifold” of fixed points of the flow. This is related to the concept of “timescale separation” [20] within complex systems, whereby one component of the system is undergoing changes rapidly enough to be considered as jumping instantaneously between equilibria.

The vanishing lag between changes in the number of shedding, infectious hosts, and the resulting response in the force of infection is the reason why ET via short-lived pathogen can be approximated as DT. Systems with ET that results from the accumulation of relatively long-lived pathogen retain a *memory* of the size of the infectious host sub-population since hosts that have since been removed, or have ceased to shed, may still be the cause of new exposures via the pathogen that they had previously emitted. This delay between changes in the number of infectious hosts and their effect on the system dynamics violates the implicit assumption of DTMs that the force of infection is directly related to the number of infectious hosts, or the number of hosts who are shedding pathogen. As the pathogen lifetime decreases, so does this memory effect, and we see an increasing similarity with the dynamics produced by direct transmission.

**Fig 3:**
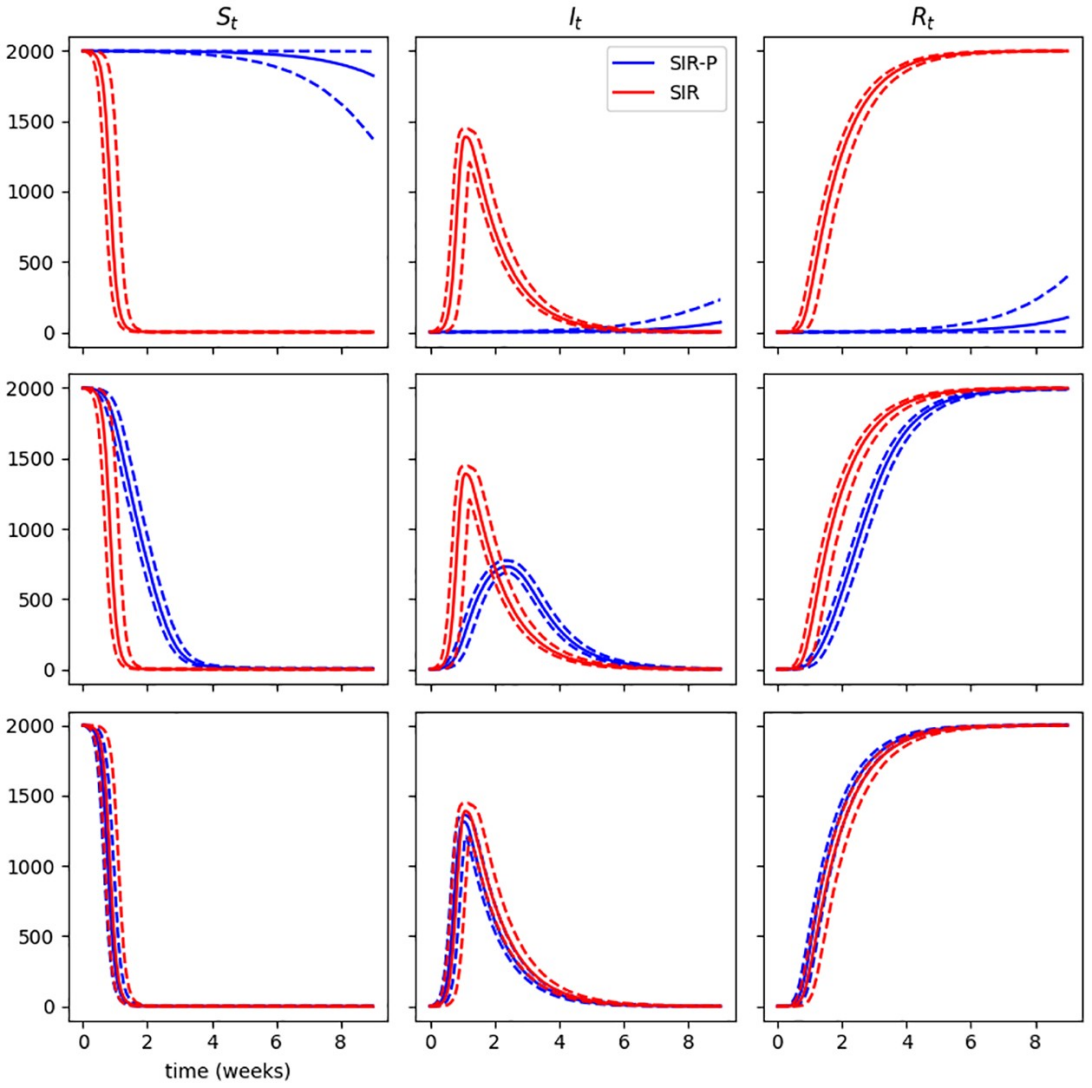
Susceptible, infectious and removed host sub-population sizes of SIR-P (blue) process averaged over 5000 simulations for fixed environmental transmission rate *α* = 5.95 × 10^−7^ virion^−1^ h^−1^ and host mortality rate *γ* = 5.95 × 10^−3^ h^−1^. The rates of pathogen emission, *ϵ*, and pathogen removal, *ρ*, are increased while keeping their ratio fixed, $\frac{\epsilon }{\rho }=50.0$. **Top row**: *ϵ* = 2.98 × 10^−2^ host^−1^ h^−1^, *ρ* = 5.95 × 10^−4^ h^−1^, **middle row**: *ϵ* = 2.98 host^−1^ h^−1^, *ρ* = 5.95 × 10^−2^ h^−1^, **bottom row**: *ϵ* = 2.98 × 10^2^ host^−1^ h^−1^, *ρ* = 5.95 h^−1^. For comparison, the same sub-population sizes for the DTA SIR process with fixed direct transmission rate $\beta =\alpha \frac{\epsilon }{\rho }=2.98\times 10^{-1}{\text{host}}^{-1}{\text{h}}^{-1}$ and *γ*′ = 5.95 × 10^−3^ h^−1^ are plotted in (red). Median population sizes indicated by bold lines, dashed lines indicate 5^th^ and 95^th^ percentiles. The top row of panels show two processes that are visibly distinct in their outputs, but with a hundred-fold increase in the pathogen decay rate, a closer alignment between the two sets of trajectories can be seen in the middle row. In the last case (bottom row) there is no difference on the scale of the plots between the SIR-P and SIR model outputs.

Fig 3 demonstrates this behaviour by showing susceptible, infectious and removed sub-population sizes averaged over a number of independent simulations from three SIR-P models with fixed *α* and *γ* and increasing *ϵ* and *δ*, with $\frac{\epsilon }{\rho }$ held constant. These are plotted in each case against similar outputs from a SIR model with the same *γ* and *β* chosen to equal $\alpha \frac{\epsilon }{\rho }$, so that the SIR model is the DTA of the SIR-P model. As shown in the figure, increasing rates of pathogen emission and decay rate lead to increasing similarity between the SIR-P and SIR model outputs, and in the case of short-lived pathogen, the SIR-P and DTA SIR outputs are indistinguishable on the scale of the plots.

## Results

### Estimating DTA parameters from outbreak data

The presence of environmental transmission violates an assumption of DTMs: that the force of infection is directly related to the number of infectious hosts currently in the system. Here we fited the DT SEIR model to data from simulated SEIR-P outbreaks with varied rates of pathogen emission and decay and assessed the goodness of fit of the model using GPPCs (Materials and methods). The simulated data consists of times of onset of infectivity and host removal, i.e., the times of entry of hosts into the I and the R states, the observation of which is feasible in some cases (see Case study: White spot disease in penaeid shrimp).

The data set specifications are summarised in Table 3 and the MCMC methods of model fitting and a description of the GPPCs are found in Materials and methods and Section A in S1 Appendix. The first three data sets were generated using a SEIR-P process in which there was both environmental transmission due to pathogen as well as direct transmission from host to host. The rates of pathogen emission, *ϵ*, and decay, *ρ*, were increased with $\frac{\epsilon }{\rho }$ kept constant. The fourth data set has no environmental transmission rate, and so is the output of a DTM.

**Table 3:** Scenarios for simulation study.

Data generating process	parameter values	N	m
A. *long-lived pathogen*	*α* = 0.001 virion^−1^ d^−1^, *β* = 0.007 host^−1^ d^−1^	300	295
	*I*_*j*_ − *E*_*j*_ ∼ Gamma(1.10, 0.5 d^−1^)		
	*R*_*j*_ − *I*_*j*_ ∼ Gamma(1.10, 1.0 d^−1^)		
	*ϵ* = 5.4 virion host^−1^ d^−1^, *ρ* = 0.8 d^−1^		
B. *intermediate pathogen*	*α* = 0.001 virion^−1^ d^−1^, *β* = 0.007 host^−1^ d^−1^	300	294
	*I*_*j*_ − *E*_*j*_ ∼ Gamma(1.10, 0.5 d^−1^)		
	*R*_*j*_ − *I*_*j*_ ∼ Gamma(1.10, 1.0 d^−1^)		
	*ϵ* = 54.0 virion host^−1^ d^−1^, *ρ* = 8.0 d^−1^		
C. *short-lived pathogen*	*α* = 0.001 virion^−1^ d^−1^, *β* = 0.007 host^−1^ d^−1^	300	297
	*I*_*j*_ − *E*_*j*_ ∼ Gamma(1.10, 0.5 d^−1^)		
	*R*_*j*_ − *I*_*j*_ ∼ Gamma(1.10, 1.0 d^−1^)		
	*ϵ* = 5.4 × 10^4^ virion host^−1^ d^−1^, *ρ* = 0.8 × 10^4^ d^−1^		
D. *direct transmission only*	*α* = 0.0 virion^−1^ d^−1^, *β* = 0.0075 host^−1^ d^−1^	300	272
	*I*_*j*_ − *E*_*j*_ ∼ Gamma(1.10, 0.5 d^−1^)		
	*R*_*j*_ − *I*_*j*_ ∼ Gamma(1.10, 1.0 d^−1^)		

Total host population size = *N*. Final outbreak size = *m*.

#### MCMC convergence and posterior coverage

Convergence of the MCMC sampling chains was checked by running two separate chains for each fitted model with separated initial values and observing that they converge to a common stationary distribution. Trace plots for the two parameters that were MCMC sampled, *β* and *δ* can be found in Fig A in S1 Appendix.

**Fig 4:**
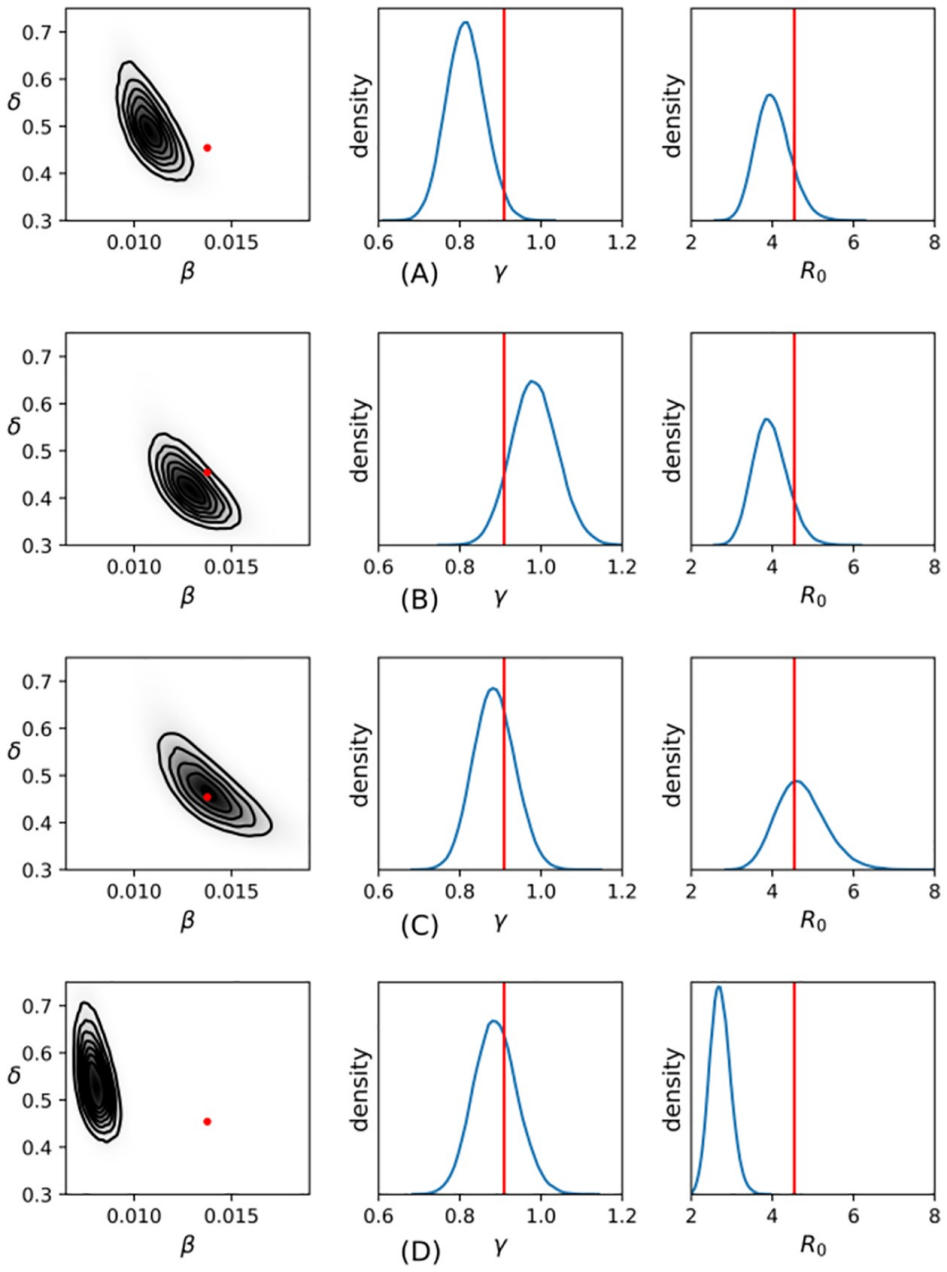
Density estimates of SEIR parameter posterior distributions and *R*_0_ for long-lived pathogen (A), intermediate pathogen (B), short-lived pathogen (C) and direct-transmission only (D) data sets. The red dot in the leftmost panels indicates $\left(\hat{\beta },\hat{\delta }\right)$, where $\hat{\beta }=\alpha \epsilon /\rho +\beta$ and $\hat{\delta }={\left(E(I_{j}-E_{j})\right)}^{-1}$. The red vertical lines in the central and rightmost panels indicate $\hat{\gamma }={\left(E(R_{j}-I_{j})\right)}^{-1}$ and $\hat{R_{0}}=\frac{\hat{\beta }N}{\hat{\gamma }}$, respectively. The marginal posterior distributions *γ* and (*β*, *δ*) are conditionally independent and so are plotted separately.

Fig 4 is a graphical summary of the samples obtained from the posterior distribution of the parameters and of the SEIR basic reproduction ratio (the expected number of new cases of infection that result from addition of a single infected host into a large, wholly susecptible population), $R_{0}=\frac{\beta N}{\gamma }$, for each of the fitted models. For comparison, these are plotted against the reference values $\hat{\beta },\hat{\delta },\hat{\gamma }\;\text{and}\;{\hat{R}}_{0}$, where $\hat{\beta }=\alpha \frac{\epsilon }{\rho }+\beta$ is the effective transmission rate defined in The direct transmission approximation as timescale limit of SEIR-P process. The quantity $\hat{\delta }=E{\left(t^{I}-t^{E}\right)}^{-1}$ and the quantity $\hat{\gamma }=E{\left(t^{R}-t^{I}\right)}^{-1}$ are, respectively, the reciprocals of the mean E and I lifetimes in the underlying process and ${\hat{R}}_{0}=\frac{\hat{\beta }N}{\hat{\gamma }}$ is the basic reproductive ratio for the SEIR-P process, according to the survival function formulation (see, e.g., [21, 22]).

The position of $\hat{\beta },\hat{\delta }\;\text{and}\;\hat{\gamma }$ close to the centre of the parameter posterior distribution in the short-lived pathogen case in Fig 4C means that a SEIR process with direct transmission rate $\hat{\beta }=\alpha \frac{\epsilon }{\rho }+\beta$ and (exponentially-distributed) E and I lifetimes with means that match those of the underlying process is the most likely model from among the class of SEIR models with exponential lifetimes. As a result, the fitted model produces a very good estimate of *R*_0_ in relation to the underlying SEIR-P process.

As the pathogen lifetime increases in Fig 4A and 4B, leading to a lesser degree of timescale separation, the fitted models both underestimate *R*_0_ and $\hat{\beta },\hat{\delta }\;\text{and}\;\hat{\gamma }$ lie further from the centre of the parameter posterior distribution (in the tails in the long-lived pathogen case).

#### Assessing DTA model fit against ET outbreak

**Fig 5:**
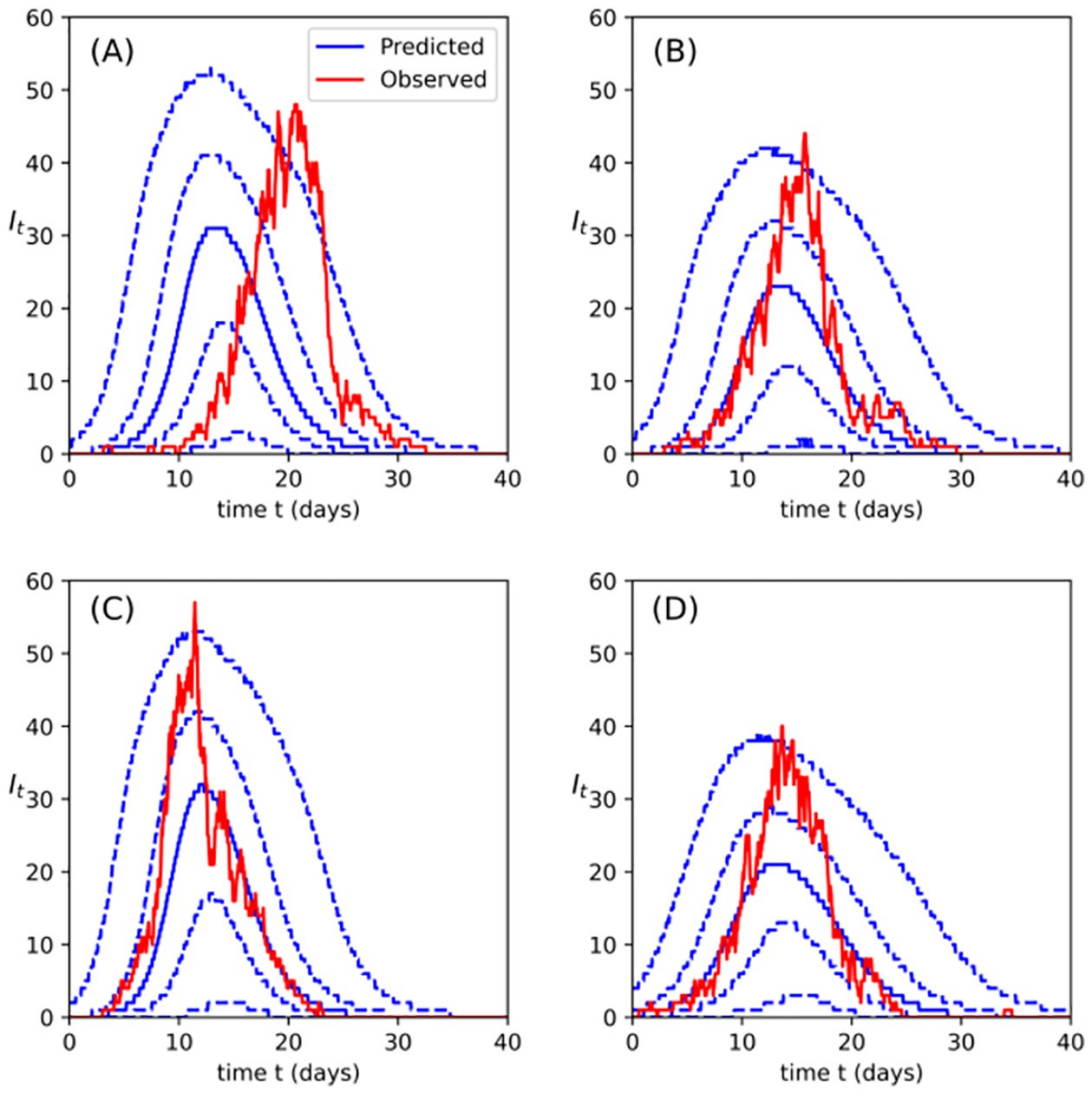
Observed outbreak size trajectories, *I*_*t*_, over course of a single simulated outbreak (solid red line): long-lived (A), intermediate (B) and short-lived pathogen (C) and direct transmission only (D). These are compared with the trajectories obtained from SEIR model with MCMC-sampled parameters values, with *small* outbreaks (≤ 50) discarded. The time axis was discretised (400 points) and 5^th^, 25^th^, 50^th^ (median), 75^th^ and 95^th^ percentiles of the SEIR-predicted outbreak size were estimated at each discrete time point. The solid blue line indicates the median outbreak size while the dashed blue lines are the other percentiles. In the short-lived and direct transmission only cases, the shape of the predicted outbreak size trajectories (as indicated by the blue lines) mirrors that of the observed outbreak size, with the solid red and blue lines aligning at the initial exponential growth phase, as well as at the end of the outbreak when the number of infective hosts dies out. This is not the case for the long-lived pathogen case, for which the model predicts earlier onset of growth of the outbreak, peaking somewhat earlier than was observed.

First, we compare the outbreak size trajectories predicted by the fitted model with the trajectory obtained from the data. These were obtained by simulating SEIR event times with parameter values drawn from the posterior samples, as discussed in Materials and methods. If the model fits well then we should expect these posterior-predicted trajectories to look similar to the observed trajectory [23, 24]. Fig 5 graphically compares the posterior-predictive outbreak size trajectories for the four cases described above with the underlying observed outbreak trajectory. The outbreak size trajectory from the data is indicated by the solid red line and superposed on this is a graphical summary of posterior predictive outbreak size trajectories. The solid blue line indicates the median predicted number of infectious hosts, while the blue dashed lines are the 5^th^, 25^th^, 75^th^ and 95^th^ percentiles. In the cases of intermediate and short-lived pathogen, the predicted model output appears to agree well with the data, as is the case with DT only. However, for the long-lived pathogen, the model appears to predict outbreaks that reach their peak and begin to recede sooner than was observed in the data. Nonetheless, the fitted model does agree with the data in terms of peak outbreak size.

**Fig 6:**
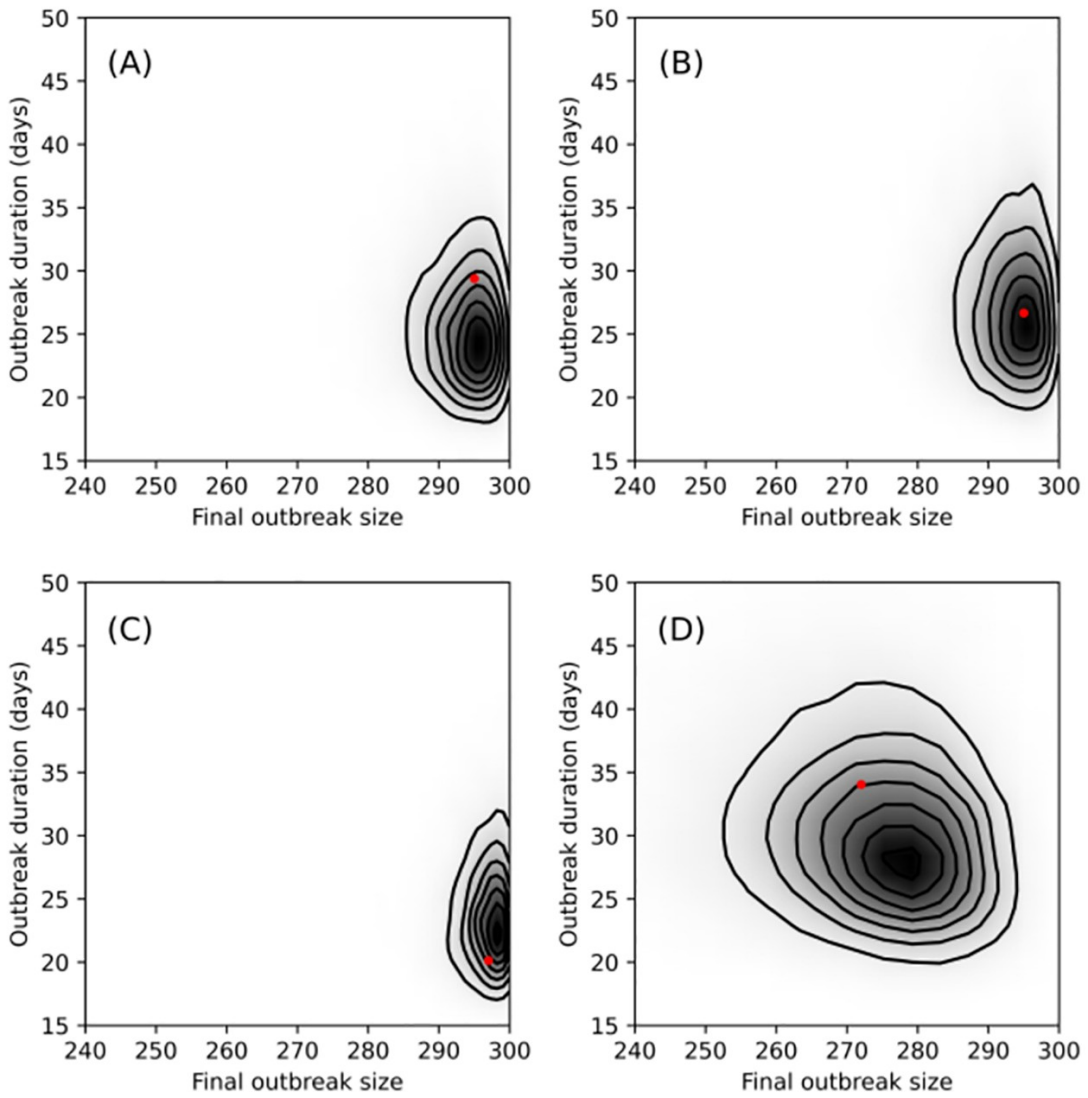
Graphical comparisons of final outbreak size (total hosts infected during outbreak) vs. outbreak duration (latest removal time minus time of first onset of infectivity) with their posterior predictive distributions. The red dot indicates observed value of statistics from one outbreak: long-lived (A), intermediate (B) and short-lived pathogen (C) and direct transmission only (D). The shading and contours were obtained from a kernel density estimate after simulating 15000 SEIR outbreak trajectories with parameter values taken from the MCMC samples obtained while fitting the SEIR model, with *small* outbreaks (≤ 50) discarded. In the case of long-lived pathogen, the fitted model tends to predict shorter duration outbreaks but otherwise agrees with the data in terms of final outbreak size. This is indicated by the red dot aligning horizontally with the darkest part of the density estimate but being shifted vertically. Better agreement between the data and fitted model is evident in the short-lived and intermediate pathogen and DT-only cases.

**Fig 7:**
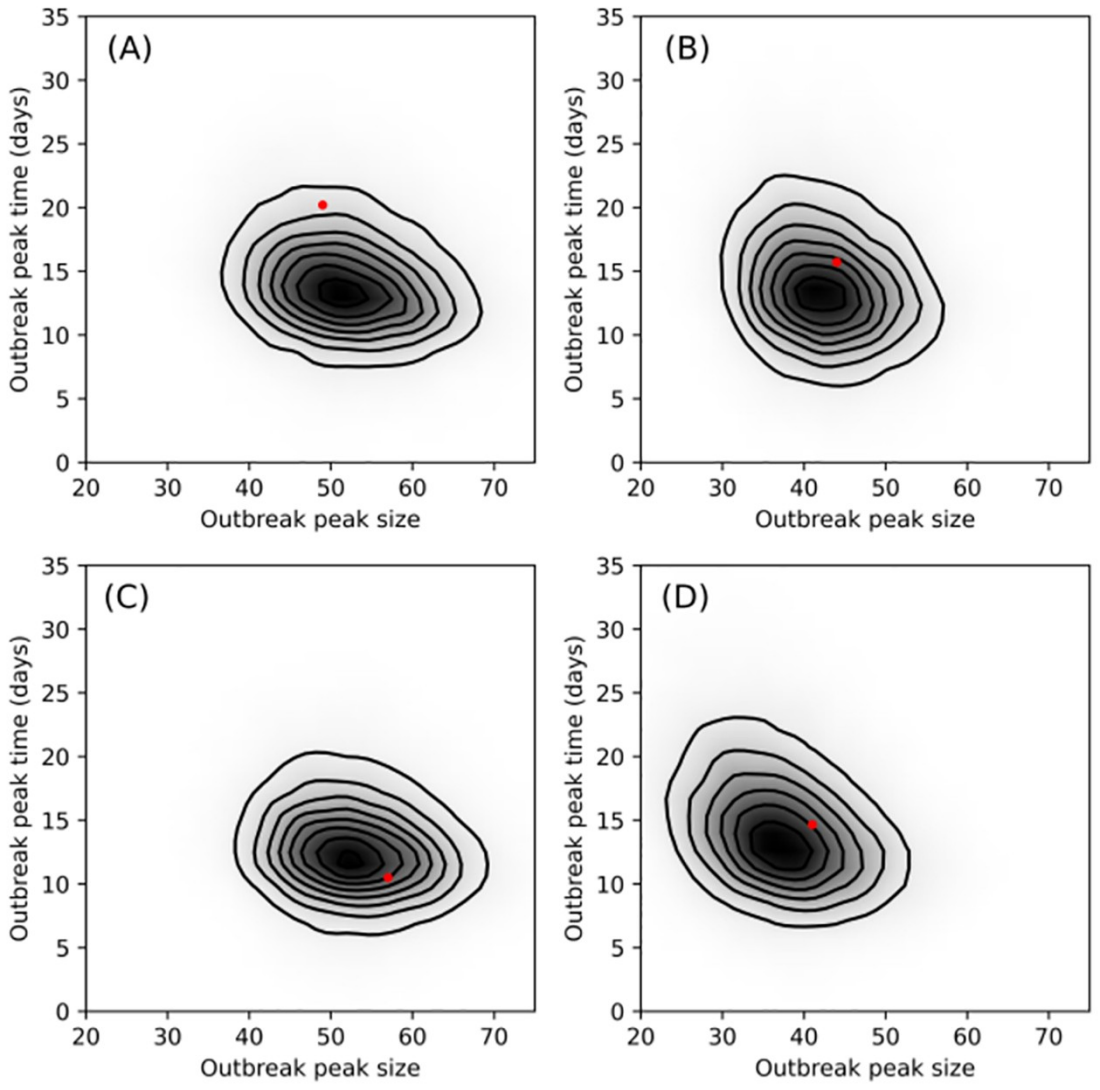
Graphical comparisons of size of outbreak peak, i.e., the size of *I*_*t*_ at its largest, and time of outbreak peak, as defined in main body of text with their posterior predictive distributions. The red dot indicates observed value of statistics from one outbreak: long-lived (A), intermediate (B) and short-lived pathogen (C) and direct transmission only (D). The shading and contours were obtained from a kernel density estimate after simulating 15000 SEIR outbreak trajectories with parameter values taken from the MCMC samples obtained while fitting the SEIR model, with *small* outbreaks (≤ 50) discarded. For long-lived pathogen, the fitted SEIR model predicts that outbreaks peak, on average, at the size observed in the data. However, the model predicts outbreaks that peak *earlier*. This is evident in panel (A), where the predicted outbreak size trajectories clearly peak earlier than the observed outbreak size trajectory indicated by the solid red line. Better agreement between data and model predictions are visible in panels (C) and (D).

Fig 6 compares graphically the final outbreak size and the outbreak duration associated with each of the four sets of onset of infectivity and removal times with the same quantities drawn from their respective posterior-predictive distributions. Fig 7 is similar, comparing size and time of outbreak peak (i.e., the outbreak at its largest) (see Materials and methods for details). As is indicated clearly in the plots, for the long-lived pathogen case, the model makes a poor prediction of when the outbreak peaks and the how long the outbreak persists before finally dying out. Better agreement is evident for the intermediate and short-lived pathogen, more so, in fact, than is evident in the DT only case.

## Case study: White spot disease in penaeid shrimp

In this section we focus on white spot disease (WSD) in penaeid shrimp, since this is a key example of an infectious disease transmitted via both DT and ET due to a pathogen known to be long-lived in the environment, under the right conditions. We formulate a SEIR-P model of the WSD-penaeid system in which attempts are made at regular intervals to remove dead and diseased hosts from the system. We estimate its parameters from published data and then compare the effect upon the outbreak trajectories resulting from stepping up the frequency of removals from every 24 h to every 6 h.

This increase in the frequency of removals is an example of how relative shortening of the SEIR-P timescales might come about in practice. We see that with removals every 24 h the SEIR-P host-disease dynamics are closely replicated by its direct transmission approximation (DTA), whereas SEIR-P and DTA visibly diverge in their averaged outputs with removals every 6 h.

### Background: WSD and its impact

WSD is a devastating viral disease caused by the white spot syndrome virus (WSSV) that affects a wide host range, including penaeid shrimp, such as the Asian tiger shrimp, *Penaeus monodon* and the whiteleg shrimp, *Litopenaeus vannamei*. Disease onset and mortality occur in shrimp quickly after exposure, with 90% of farmed stocks typically lost to disease within 2 d to 7 d [25] and nearly 100% mortality of experimentally infected shrimps observed after 5 d to 7 d [26]. Under laboratory conditions, WSSV has been shown to retain its infectivity in sea water for up 12 days and in sun dried and water-logged pond sediment for up to 19 and 35 days, respectively [27]. Through periodic sampling of seawater from abandoned shrimp culture ponds and surrounding canals in Vietnam, where previously an outbreak of WSD had led to 100% mortality of cultivated shrimp, the authors of [28] found that WSSV remained detectable for up to 20 months. The detection rates declined throughout the duration of the study with the steepest declines observed between July and December of both 2001 and 2002. The authors suggest this is linked to decreased plankton biomass during that period, which in turn suggests that WSSV is able to replicate within certain plankton species. Esparza-Leal, et.al. [29] suggest that free-floating WSSV virions have the potential to infect shrimp in pond water at around 27°C, whereas pond water temperatures in range of 30–33°C prohibit infection. In [29] it was noted that detectability of WSSV varied among pond water samples taken simultaneously from the same pond, leading the authors to note a degree of stochasticity in relation to the waterborne pathogen load.

### Modelling WSD and estimation of SEIR-P parameters

Estimates of rates of WSD transmission via ingestion and cohabitation have been made by Lotz and Soto [30] for *Litopenaeus vannamei* and by Tuyen, et al [31] for both *Litopenaeus vannamei* and *Penaeus monodon*. Each of these estimates rely on the assumption that the force of infection is proportional to the number of infected shrimp currently present in the tank (*I*_*t*_) and therefore responds immediately to changes in this number.

Tuyen, et al, decompose the rate of direct transmission into two parts arising from ingestion and cohabitation, *β* = *β*_ingest_ + *β*_cohab_ (our notation), so that the force of infection at time *t* is $\frac{\beta I_{t}}{N}$ (where they assume that transmission is frequency dependent). They estimate the two components of *β* using regression analysis of data obtained via an immersion challenge experiment where the relative amounts of exposure via the two routes are controlled. This is done for *Litopenaeus vannamei* and *Penaeus monodon* independently, and for both species combined.

Lotz and Soto expose *Litopenaeus vannamei* to WSSV exclusively via either ingestion or cohabitation for a set duration and estimate ${\tilde{\beta }}_{\text{ingest}}\;\text{and}\;{\tilde{\beta }}_{\text{cohab}}$ from the numbers of shrimp that later developed the disease. Using a Reed-Frost model of epidemics (e.g., see [32]), the latter two quantities are probabilities of disease transmission per distinct susceptible-infected shrimp pair during a time interval of duration Δ*t*. The force of infection here is approximately $\frac{\tilde{\beta }I_{t}}{\Delta t}$ (see Section B in S1 Appendix), where $\tilde{\beta }$ is either ${\tilde{\beta }}_{\text{ingest}}\;\text{or}\;{\tilde{\beta }}_{\text{cohab}}$. Lotz and Soto found ${\tilde{\beta }}_{\text{cohab}}$ to be not significantly different from zero in a first experiment and to be over an order of magnitude smaller than ${\tilde{\beta }}_{\text{ingest}}$ when the experiment was repeated (${\tilde{\beta }}_{\text{ingest}}=0.56,{\tilde{\beta }}_{\text{cohab}}=0.02$). Such a relatively low rate of transmission due to cohabitation led the authors to omit this from their model of WSD in *Litopenaeus vannamei*, described in [33]. Tuyen, et al, found a similar result in the case of Penaeus monodon (*β*_ingest_ = 0.22 h^−1^, *β*_cohab_ = 0.0026 h^−1^) but for *Litopenaeus vannamei* they in fact found that the reverse was true, in that the rate of transmission via cohabitation was greater than via ingestion (*β*_ingest_ = 0.0038 h^−1^, *β*_cohab_ = 0.018 h^−1^).

Underlying the estimates of *β*_cohab_ and ${\tilde{\beta }}_{\text{cohab}}$ above is the assumption that the force of infection responds without delay to a change in the number of infected shrimp, *I*_*t*_. This assumption is indeed valid across a wide variety of cases in which the size of the environmental pathogen load responds more or less rapidly to changes in *I*_*t*_, as discussed in the previous section. However, given the slow rate of decay of infectivity of WSSV and its persistence in water bodies long after outbreaks have occurred, it is perhaps fruitful to consider the relationship between environmental pathogen load and the rate of environmental transmission as described, for example, by the SEIR-P model described in Models for direct and environmental transmission of disease. For example, Wang, et al. (2012) [34] find that an environmental transmission model similar to SEIR-P of avian flu among duck populations was able to account for the complex periodic outbreak patterns of the disease over long time periods.

Whereas Lotz and Soto and Tuyen, et al. characterise the route of transmission due to cohabitation as being implicitly direct, with rate *β*_cohab_, (since its rate is directly proportional to the number of infectious hosts), we aim here instead to characterise this as environmental transmission with rate *α*, as described in The direct transmission approximation as timescale limit of SEIR-P process. As far as we know, there is no published estimate of this quantity for WSD among penaeids. We obtain a lower estimate of *α*_L_ = 10^−4^ ml virion^−1^ h^−1^ for *Penaeus monodon*, along with an upper estimate of the pathogen decay rate, *ρ*_U_ = 0.005 h^−1^, from the results of the WSSV viability in seawater experiment by Kumar, et al., ([27], details in Section C in S1 Appendix). Lotz and Soto use a shrimp density of 12 animals per square metre of water surface in their experiment in order to mimic densities of wild populations [30]. In the simulation study, described below, we adopt a nominal water volume of 46.2 m^3^ and water surface area of 77 m^2^ to obtain a similar host density with 1000 shrimps initially in the system. Since the estimates of *α* and *β* both have dimensions volume × virion^−1^ × time^−1^ and volume × host^−1^ × time^−1^, respectively, we scale these by this nominal volume before carrying out the simulations.

The pathogen emission rate, *ϵ*, is chosen from within a range of known shedding rates for waterborne viruses (e.g., see [35, 36]). Since each dead shrimp contributes $\frac{\epsilon }{\rho }$ to the environmental pathogen load, at equilibrium, and therefore $\alpha \frac{\epsilon }{\rho }$ to the force of infection via environmental transmission, we choose a direct transmission rate $\beta =10\times \alpha \;\frac{\epsilon }{\rho }$. This is in accord with the relative sizes of Lotz and Soto’s estimates of ${\tilde{\beta }}_{\text{ingest}}\approx 10\times {\tilde{\beta }}_{\text{cohab}}$.

**Fig 8:**
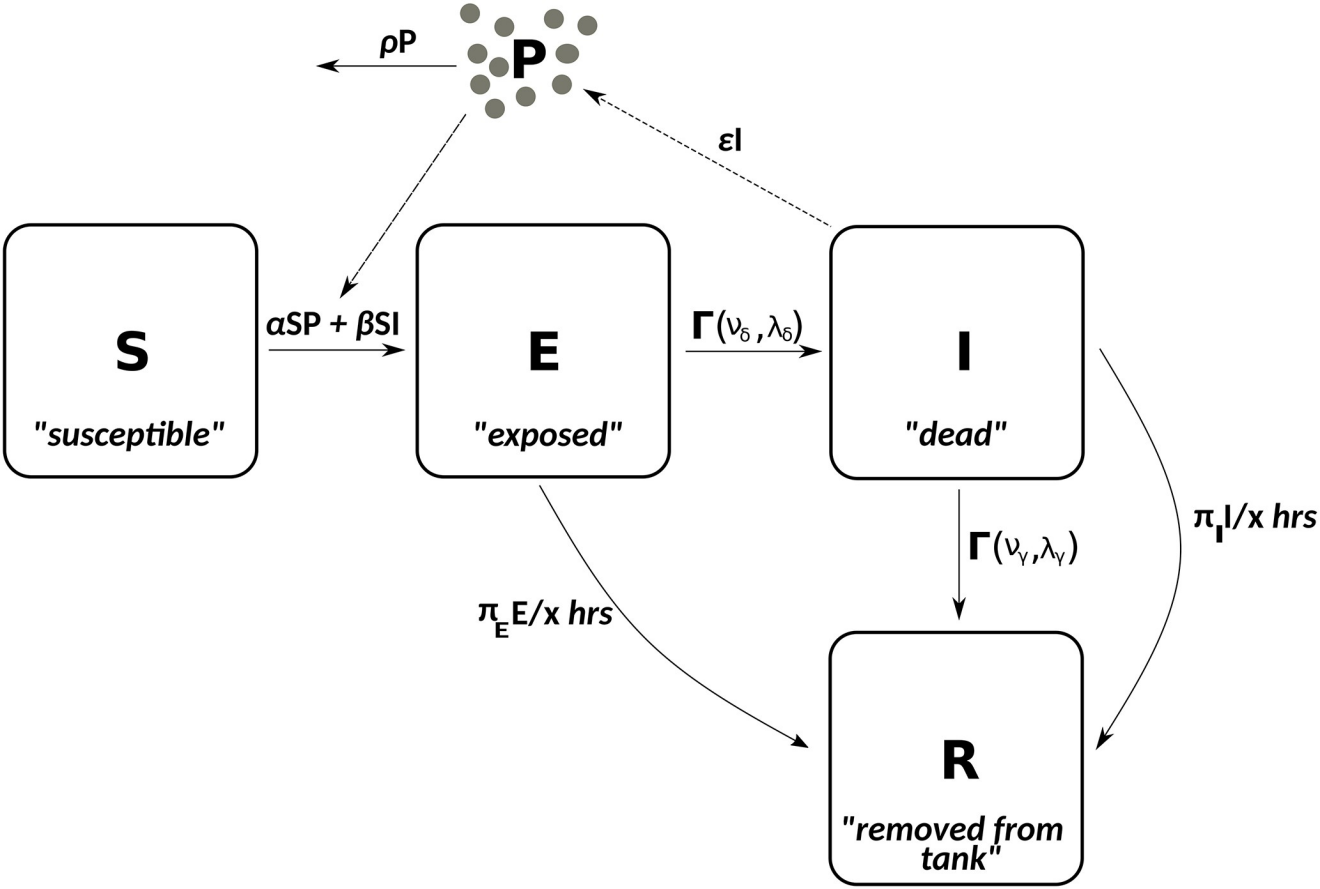
Summary of SEIR-P model of WSD among penaeids. Parameter values are listed in Table 4. The arrow from I to R, labelled Γ(*ν*_*γ*_, λ_*γ*_), represents removal of dead hosts after a gamma-distributed time to full natural decay. The curved arrow from I to R represents removal at one of the x-hourly removal attempts, with probability *π*_*I*_, similarly for the curved arrow from E to I. The *direct transmission approximation* (DTA) of the SEIR-P model is obtained by replacing *αS*_*t*_*P*_*t*_ + *βS*_*t*_*I*_*t*_ above the leftmost arrow with $\hat{\beta }=\alpha \frac{\epsilon }{\rho }+\beta$ and setting *ϵ* = *ρ* = 0.

The S, E, I and R compartments of the SEIR-P model (summarised in Fig 8) are, respectively, shrimp that are susceptible (S), have been exposed, but still alive (E), dead, and now causing new infections either via shedding virus into the water body due to decay or being scavenged upon (I) and physically removed from the system (R). We assume for simplicity that there is no viral shedding or infectivity during the E stage and that times from exposure to mortality are gamma-distributed with shape and rate parameters *ν*_*δ*_ and λ_*δ*_ such that the mean time from exposure to mortality ($\frac{\nu_{\delta }}{\lambda_{\delta }}$) agrees with the estimate given by [31].

There are two processes by which shrimp are removed from the system. Firstly, there is the long process of decay characterised by gamma-distributed times in the I compartment, with shape and rate parameters *ν*_*γ*_ and λ_*γ*_ with mean $\frac{\nu_{\gamma }}{\lambda_{\gamma }}=333.3\;\text{h}\approx 14\;\text{d}$. Secondly, removals occur probabilistically from both the E and I compartments at regularly spaced time points with probabilities of success of *π*_*E*_ = 0.05 and *π*_*I*_ = 0.95, respectively, so that bin(*E*_*t*_, *π*_*E*_) and bin(*I*_*t*_, *π*_*I*_) shrimp are removed at each removal point, *t*. Shrimp that are dead are therefore removed at the first removal time, post-mortem, with probability 0.95 and at the second with probability 0.9975. This means that it is highly unlikely that shrimp are removed from the system due to natural decay in this scenario. We assume that no living, disease-free shrimp are accidentally removed in this process. All of these model quantities are summarised together in Table 4.

**Table 4:** Parameter estimates and sources for SEIR-P model of WSSV in penaeids.

	Description		source / comment
*α*	transmission (cohabitation)	10^−4^ ml virion^−1^ h^−1^	estimated from [27] (Section C in S1 Appendix)
		2.16 × 10^−12^ virion^−1^ h^−1^	scaled by 46.2 m^3^
*β*	transmission (ingestion)	8.64 × 10^−4^ shrimp^−1^ h^−1^	$10\times \alpha \frac{\epsilon }{\rho }$ (see [30] and above discussion)
$\hat{\beta }$	direct transmission (DTA)	9.5 × 10^−4^ shrimp^−1^ h^−1^	$\alpha \frac{\epsilon }{\rho }+\beta$
*ν*_*δ*_	mortality (shape)	1.5	
λ_*δ*_	mortality (rate)	0.0112 h^−1^	[31]
*ν*_*γ*_	removal (decay) (shape)	2.0	
λ_*γ*_	removal (decay) (rate)	0.006 h^−1^	
*π*_*E*_	success of removal (from E)	0.05	
*π*_*I*_	success of removal (from I)	0.95	
*ϵ*	WSSV shedding	2 × 10^5^ virion shrimp^−1^ h^−1^	(see e.g. [35, 36])
*ρ*	loss of WSSV infectivity	0.005 h^−1^	estimated from [27] (Section C in S1 Appendix)

### Impact of removal frequency on WSD outbreaks among penaeids

**Fig 9:**
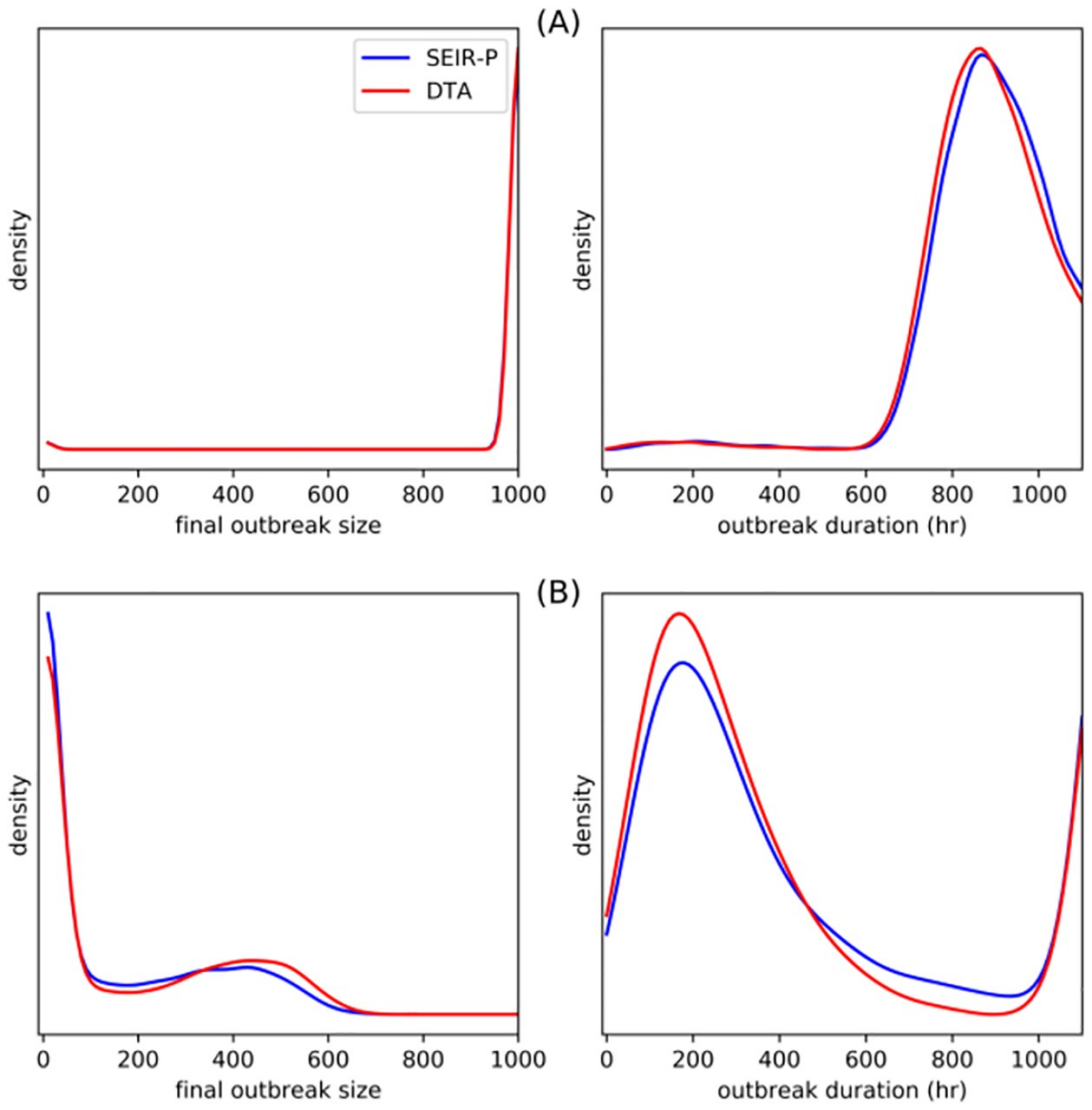
Estimated density plots of final outbreak size (left panels) and outbreak duration (right) for the SEIR-P (blue) and DTA (red) models of WSD under (A) 24-hourly removals, where both quantities are distributed very similarly under the two models, and (B) 6-hourly removals. Increasing the removal frequency tends to reduce the size and duration of outbreaks, although some larger outbreaks still occur. The benefit of increasing the removal frequency, in terms of reduction in mean final outbreak size, is underestimated slightly by the DTA and the reduction in outbreak duration is over-estimated.

**Fig 10:**
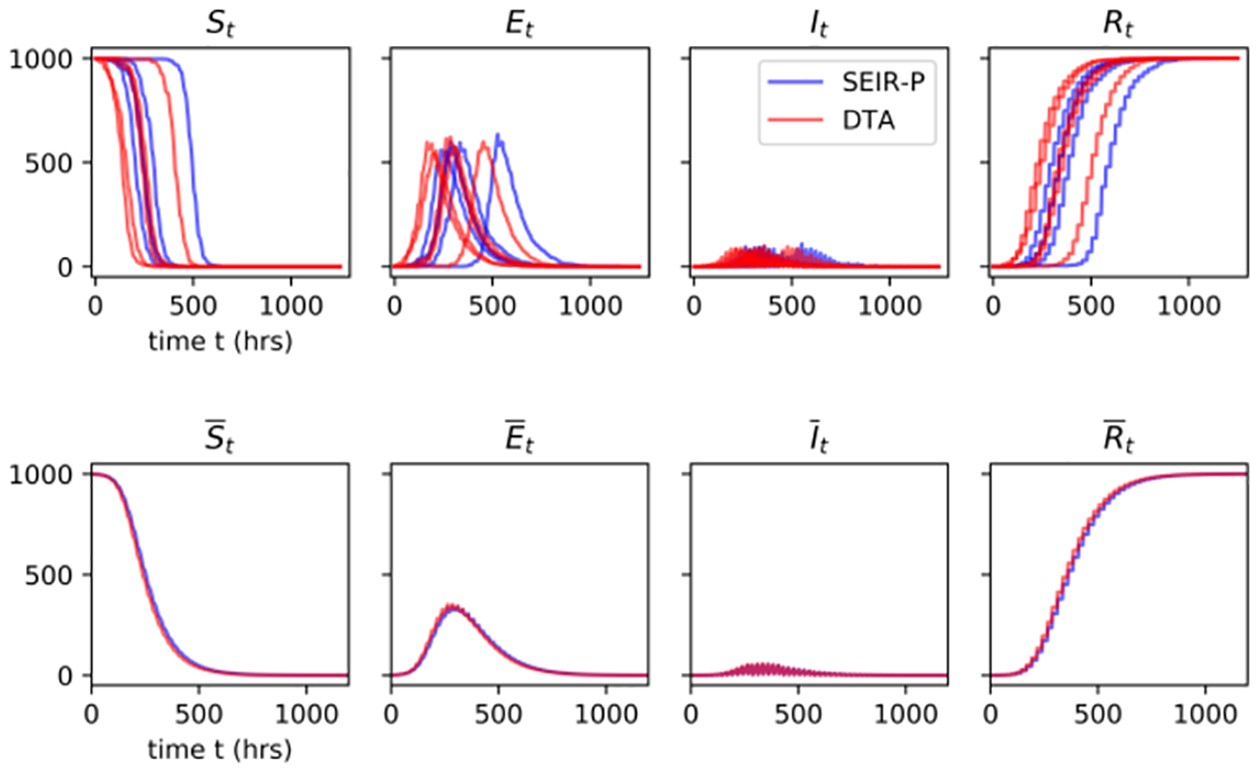
Simulations of the SEIR-P (blue) and DTA (red) models of WSD in penaeid shrimp with removals of exposed (E) and dead (I) hosts at 24-hourly intervals, with probabilities of success 0.05 and 0.95, respectively. Single outbreak trajectories (top row) and averages over 30 000 independent simulations with small outbreaks (fewer than 10 infections) excluded (bottom row). The zig-zag pattern in the 3^rd^ panel on the bottom row is due to the periodic removals. The averaged model outputs show a high degree of similarity between SEIR-P and DTA, meaning that at these timescales the environmental transmission of WSD can be well approximated with direct transmission among the hosts.

**Fig 11:**
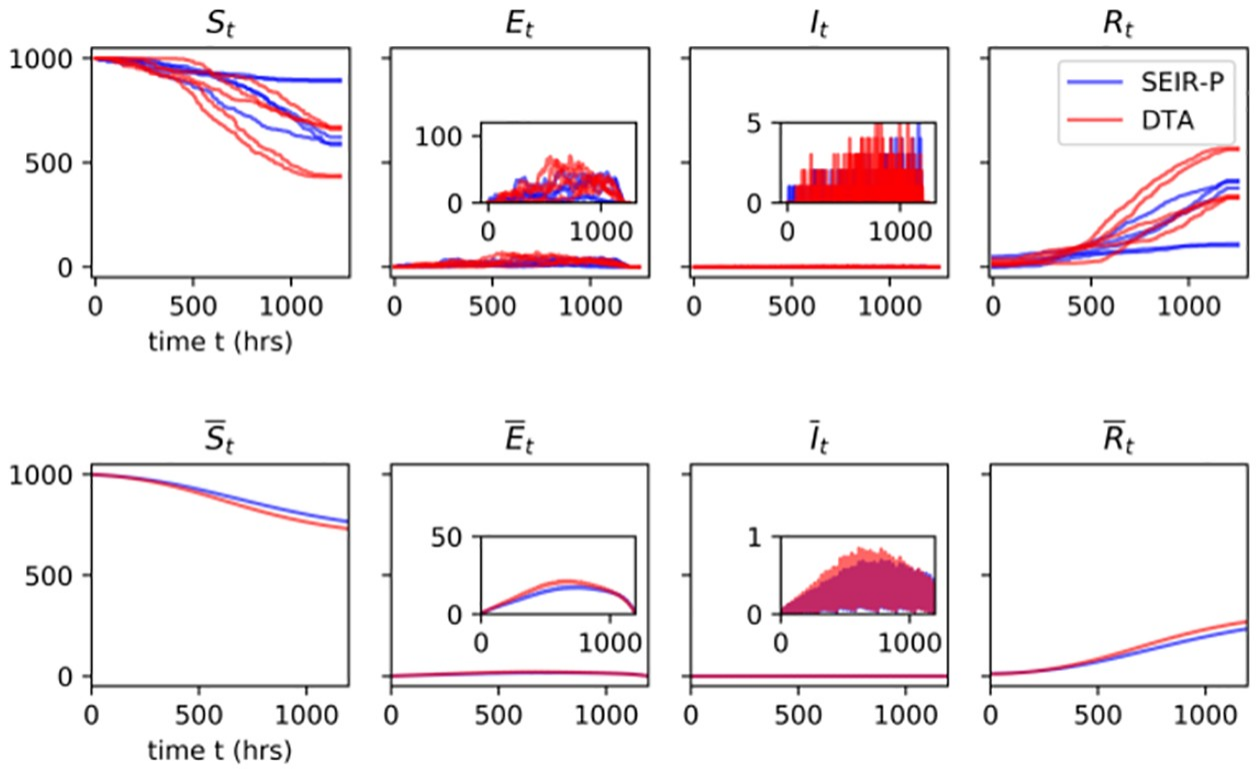
Simulations of the SEIR-P (blue) and DTA (red), as in Fig 10, with removals at 6-hourly intervals. Although the outbreaks of single SEIR-P and DTA trajectories appear similar, a small but definite divergence between the two models appears when studying their averaged outputs.

Using simulations, we study outbreak patterns under 24 and 6-hourly removals under the SEIR-P model described above. Alongside these we also look at those of the DTA of this model, where $\hat{\beta }=\alpha \frac{\epsilon }{\rho }+\beta$, for comparison. Density plots for the final outbreak size and outbreak duration, max (**t**^*R*^), of the SEIR-P model and the DTA are displayed in Fig 9 for both 24 and 6 hourly removals. Figs 10 and 11 show the simulated outbreak trajectories for the four host compartments of the SEIR-P model and DTA. The top row in these two figures are typical individual outbreak trajectories while the bottom row are trajectories averaged over 3 × 10^4^ independent simulations, with “small” outbreaks of fewer than 10 infections removed.

Figs 9A and 10 show that attempting to remove the dead shrimp from the system at 24 h intervals, even with a success rate of 95%, is not sufficient to prevent large outbreaks of WSD, with outbreaks overwhelmingly affecting more than 90% of the shrimp population and lasting more than 600 h from index exposure to final removal. Increasing the intensity of surveillance, however, by removing dead and diseased shrimp every 6 h, eliminates many such large outbreaks, limiting the final outbreak size to about 60% of the population. Additionally, outbreaks tend to be eradicted sooner, at around 200 h, although a sizeable proportion continue for longer (see Fig 9B).

It is interesting to compare the SEIR-P and DTA trajectories when going from 24 to 6-hourly removals, since the time that infectious shrimp are in the system is reduced by about a quarter, on average. This is an example of how two distinct degrees of host and pathogen timescale separation may be observed for the same host-disease system. The plots in Figs 9A and 10 suggest very close alignment between the SEIR-P and DTA models in their host compartment dynamics, final outbreak sizes and outbreak durations, meaning that we can faithfully reproduce the environmental transmission without needing to model the pathogen load. Under such a scenario, most hosts remain infectious for less than 24 h. Nonetheless, we see that the resulting outbreak patterns are captured equally well by the DTM as by the full SEIR-P model. The shortening of the host timescale by removing every 6 h is sufficient, however, to begin to observe divergence between the SEIR-P and the DTA, most noticeably, perhaps, in the distributions of the final outbreak size and outbreak duration (Fig 9B). Indeed, the DTA underestimates, on average, the reduction in final outbreak size and overestimates the reduction in outbreak duration. The DTA outbreaks at 6-hourly removals tend to grow slightly faster than the SEIR-P outbreaks (see Fig 11).

## Discussion

We have seen in Results that the SIR and SEIR models approximate the host-disease dynamics arising from a combination of direct and environmental transmission, as modelled by SIR-P or SEIR-P, that this approximation improves with increasing rates of pathogen shedding and decay and that when fitting these models to data, using Bayesian inference and data augmentation, they are highly robust to violations of the assumption of direct transmission. For example, these results suggest that the direct transmission approximation will be suitable for modelling transmission of SARS-CoV-2 within a closed environment, such as a hospital, since it has a half life of about 1 h in aerosols and 1 h, 3.5 h, 5.75 h and 7 h on copper, cardboard, stainless steel and plastic surfaces [37] but the mean infectious period is considerably longer: 5 d to 11 d for asymptomatic cases, up to 4 d for presymptomatic cases [38] and about 7 d for symptomatic cases [39].

Tien & Earn, in their investigation of multiple transmission routes of cholera among humans [19], cite the rate of pathogen decay in the waterbody as the important factor in determining whether one should consider modelling the environmental and direct routes of transmission separately, or combined as one direct route. As suggested by the simulation study in Case study: White spot disease in penaeid shrimp, a viral lifetime of 200 h versus a much shorter host mean infectious lifetime of around 24 h also results in disease dynamics closely reproducible with a DTM, in spite of the low rate of pathogen decay. In this case the high rate of pathogen shedding produces sufficient host-pathogen timescale separation in order that DT provides a good, approximate description of the transmission via both direct and environmental routes. When the rates of shedding and pathogen decay are both low then we do not expect the DT approximation to work. Macro-parasite infections are one class of disease system within this grouping and our results indicate why models describing the complex host-parasite interaction, similar to those of similar to that of Anderson & May [40, 41], are often used for these systems (e.g., see [42]).

While individual outbreak trajectories appear very similar, statistical comparison over many runs reveals a strong and practically important divergence between environmental and direct transmission models of WSD among penaeids under more effective disease control (i.e., more frequent removals). The model fitting and checking in Results were done under the rather special scenario that both the times of onset of infectivity and host removal are known so as to construct the outbreak size trajectories displayed in Fig 5, which provides the clearest indication of lack of model fit in the long-lived pathogen case. However, DTMs can and have been fitted to a wide range of partial epidemic data [43, 44] and our conclusions will hold in such scenarios. Nonetheless GPPCs in general may not be the sharpest way to detect departure from direct transmission when the data from an outbreak is less complete, as is often the case. *Exposure time residuals* (ETRs) (Lau, et al [45]) could potentially yield a numerical measure of model fit. ETRs are defined, relative to some putative model, as joint functions of the data, latent variables and parameters and their joint posterior predictive density should approximate an independent uniform sample when the parameters are close to the mode of their posterior. However, in the case of there being latent variables, such as when the host event times are not fully observed, analysis of their high-dimensional posterior distribution is not straightforward. Common methods of model comparison, such as model evidence [46] and the Bayesian and the deviance information criteria (BIC & DIC) would require an alternative ETM fitted to the same data in order to make a comparison. It is an open question whether ETMs can be fitted to host-disease events without measurement of the environmental pathogen load or strong prior information about the pathogen shedding and decay rates. Tien and Earn [19] comment that even when pathogen dynamics are slow, parameters quantifying rates of environmental and direct transmission (*α* and *β*) are still unidentifiable from disease incidence data alone. Methods that measure pathogen density in the waterbody, such as polymerase chain reaction [47, 48] are therefore required in order to quantify environmental transmission from data.

Among the key assumptions of the SEIR-P model of WSD among penaeids is that the rate of pathogen shedding, *ϵ*, is constant, both across the host population and temporally within a single host over the course of their infection. There are, indeed, a few studies in which the rate of pathogen shedding has been measured in the same host at multiple time points, and these suggest that rates of pathogen emission do indeed vary over time (e.g., the investigation by Wargo, et al., of infectious hematopoietic necrosis virus shedding in juvenile rainbow trout [49] and that by Jones, et al., regarding repeated measures of respiratory viral load of SARS-CoV-2 [50]). What is gained by fitting a model with fixed shedding rate to epidemiological data is an idea of the “average” rate of shedding, both across the population and over time, even though the model’s estimates of risks at particular time points are either under or over-estimated. Another assumption is that the environmental pathogen is uniformly mixed throughout the water body. In the context of small to mid-sized tanks, this is reasonable, but when, e.g., considering the spread of infection between a local network of shrimp farming ponds then we would perhaps consider additionally incorporating a contact structure between multiple, uniformly mixed sites, as in [44]. Further complications may arise, however, in larger bodies of water where pathogen density perhaps varies spatially due to factors including water temperature or the existence of eddy currents, and temporally, due to effects of diffusion that cannot be neglected. Computational fluid dynamics, which numerically models flows of air and water, can be coupled with epidemiological models in order to incorporate uneven spatial pathogen densities and predict flows of pathogen carrying air and water currents (see, e.g., the study of the spatial pattern of affected households in the 2001 Amoy Gardens outbreak of SARS [51, 52].

Nonetheless, even simple models, as long as we can adequately quantify them from data, offer approximations of useful quantities, such as the likely size and duration of outbreaks. This work should offer reassurance to readers that direct transmission models, with their simple picture of disease transmission, remain powerful tools as their field of application grows.

## Materials and methods

### Bayesian model fitting and inference

#### SEIR posterior, likelihood and prior densities

In Results we fit the SEIR model with exponential exposed and infectious lifetimes to data generated by both the SEIR and SEIR-P processes. Since in practice, the times of host exposure, $t^{E}=\left\{t_{1}^{E},\ldots ,t_{m}^{E}\right\}$, are very rarely observed directly, these are treated as *missing data*. It is often the case that that the times of onset of infectivity, $t^{I}=\left\{t_{1}^{I},\ldots ,t_{m}^{I}\right\}$, are also unobservable. However, we will be using the SEIR and SEIR-P models to describe WSSV in penaeid shrimp (see Case study: White spot disease in penaeid shrimp), for which onset of infectiousness coincides roughly with the death of the shrimp. Therefore, in this particular case, the times of entry into the I state, corresponding to death (and onset of infectiousness), and the R state, corresponding to removal from the system (either due to complete decay or physical removal from the waterbody) are feasibly observable.

Bayesian inference regarding the parameters of the SEIR model is based entirely on the *posterior density*, which in the case of exponentially distributed exposed and infectious lifetimes is

$$\begin{array}{c} p\left(\;\beta ,\delta ,\gamma ,t^{E}\;|\;t^{I},t^{R}\;\right)\propto p\left(\;t^{E},t^{I},t^{R}\;|\;\beta ,\delta ,\gamma \;\right)\;p\left(\;\beta ,\delta ,\gamma \;\right) \end{array}$$ (5)

where **t**^*I*^ and $t^{R}=\left\{t_{1}^{R}<\ldots <t_{m}^{R}\right\}$ are the observed times of onset of infectivity and removal and **t**^*E*^ are the unobserved times of exposure (with indices corresponding to hosts, so that the host that is exposed at time $t_{i}^{E}$ becomes infectious at $t_{i}^{I}$ and is removed at $t_{i}^{R}$). The symbol *m* denotes the final outbreak size. The two factors on the right hand side are respectively the *likelihood* and the *prior* densities. The prior density summarises our knowledge and uncertainty about the parameters *prior* to observing the data. Throughout, we will assume that the three parameters are *a priori* independent, i.e.,

$$\begin{array}{c} p(\;\beta ,\delta ,\gamma \;)=p(\;\beta \;)p(\;\delta \;)p(\;\gamma \;) \end{array}$$ (6)

and that

$$\begin{array}{c} p(\;\beta \;)∼\text{Exp}(\omega_{\beta })\\ p(\;\gamma \;)∼\text{Exp}(\omega_{\gamma })\\ p(\;\delta \;)∼\text{Exp}\left(\omega_{\delta }\right)\;\text{or}\;\text{U}\left(0,10\right) \end{array}$$ (7)

We follow [43] in choosing exponentially-distributed priors since their functional form leads to conventient expressions for the full conditional distributions of the parameters, making it easier to sample from the posterior distribution. We choose the values *ω*_*β*_, *ω*_*δ*_ and *ω*_*γ*_ = 0.001 for each marginal prior distribution’s rate parameter so that each has a mean 1 × 10^3^ and variance 1 × 10^6^ (in appropriate units) and the resulting prior density is approximately flat over a large area of parameter space. This means that almost all of the information expressed by the posterior distribution comes from the likelihood. This kind of prior is described as *uninformative* since it expresses, in probabilistic language, that we have minimal knowledge about the values of the parameters prior to observing the data. In circumstances where we are fitting models to real data, without prior knowledge about the parameters of the model, it is necessary to ensure that a specific choice of uninformative prior is not unduly influential on posterior inferences. We perform such a *sensitivity analysis* by refitting the same model with several distinct, uninformative priors, comparing the resulting posterior distribtions and checking that these are largely unaffected by choice of prior. However, since in what follows, we are fitting models to simulated data with known parameter values, we select priors in advance that we know to be sufficiently uniform on the scale of the likelihood. We will see, in what follows, that even with no prior knowledge about the direct transmission rate, *β*, (as expressed by its uninformative marginal prior density) it is possible to estimate this quantity in the absence of observed exposure times (see, e.g., [43, 44, 53]).

In the case of long-lived pathogen, SEIR is far from the “correct” model for the data (e.g., see [54, 55] for accounts of fitting mis-specified models) and this can present issues with the convergence of the MCMC chains since there are no strong candidates among parameter values that simultaneously explain the data. In order to aid convergence, therefore, the alternative, uniformly-distributed prior for *δ* is used in the long-lived pathogen case only (Estimating DTA parameters from outbreak data). By placing an upper bound (in this case, 10.0) on the support of *δ*, we stipulate that we seek a model with non-negligible latent infectious periods, of duration no shorter than one tenth of a day.

The likelihood, *p*(**t**^*E*^, **t**^*I*^, **t**^*R*^ | *β*, *δ*, *γ*), describes how the data depend on the parameters of the model, which, in the case of exponentially distributed times in the E and I states is

$$\begin{array}{cc} p(\;t^{E},t^{I},t^{R}\;|\;\beta ,\gamma ,\delta \;) & \propto \prod_{i\neq \kappa }\{\beta I_{t_{i}^{E}-}\}\times \text{exp}\{-\beta \int_{t_{\kappa }^{E}}^{\text{max}\left(t^{R}\right)}S_{t}I_{t}\;dt\}\times \cdots \\ & \cdots \times \prod_{j=1}^{m}\delta e^{-\delta (t_{j}^{I}-t_{j}^{E})}\times \prod_{j=1}^{m}\gamma e^{-\gamma (t_{j}^{R}-t_{j}^{I})}. \end{array}$$ (8)

where $I_{t_{i}^{E}-}$ is the number of infectious hosts *immediately before* the i^th^ exposure time. See Section A in S1 Appendix.

The products over *j* = 1, …*m* in (8) are the contributions to the likelihood from each of the exponentially-distributed times spent in states E and I. The terms in the product over *i* ≠ *κ* are the contributions from the exposure times, excluding the index exposure, which each have associated hazard *βS*_*t*_ *I*_*t*_.

Similarly to [43], $t_{\kappa }^{E}$ represents the (perhaps unobserved) first exposure time. In Results we fit these models in scenarios where the exposure times have not been observed and so the time of the index exposure is unknown.

For details of model fitting, see Section A in S1 Appendix.

#### Model checking using graphical posterior-predictive checks (GPPC)

Graphical posterior predictive checks (GPPC) [23, 24] are used here to test for departure from DT model assumptions. These are a standard model checking tool, offering a visual comparison of quantities derived from the observed data, *h*(**t**^*I*^, **t**^*R*^), with *h*(**t**′^*I*^, **t**′^*R*^), where **t**′^*I*^, **t**′^*R*^ are simulated from the posterior-predictive distribution of the fitted model, with density

$$\begin{array}{ll} p({t^{\prime }}^{I},{t^{\prime }}^{R}|t^{I},t^{R}) & =\int p({t^{\prime }}^{I},{t^{\prime }}^{R}|\beta ,\delta ,\gamma ,t^{E},t^{I},t^{R})p(\beta ,\delta ,\gamma ,t^{E}|t^{I},t^{R})d\beta \;d\delta \;d\gamma \;dt^{E}\\ & =\int p({t^{\prime }}^{I},{t^{\prime }}^{R}|\beta ,\delta ,\gamma )p(\beta ,\delta ,\gamma ,t^{E}|t^{I},t^{R})d\beta \;d\delta \;d\gamma \;dt^{E} \end{array}$$ (9)

Uncertainty regarding parameter values is expressed by drawing from their posterior distribution. The idea is to check that the fitted model replicates the original data with reasonable probability, with no large, systematic disagreements between the data and model predictions.

Among the salient features of a disease outbreak are its size, at both peak and completion, and characteristic timescales, e.g., time from index exposure to peak of outbreak, and total duration. Such statistics are of interest in their own right and there are known formulas in the deterministic case for peak and final outbreak sizes and initial exponential growth rates for SIR, SEIR and similar models [56–58]. For the GPPCs here we obtain a probabilistic picture of similar quantities with the timing of the outbreak’s peak standing as proxy for the initial growth rate. The following four quantities are considered.

1. final outbreak size, *m*, i.e., total hosts who become exposed during outbreak
2. outbreak duration, max (**t**^*R*^) − min (**t**^*I*^)
3. time of outbreak peak, *t*_peak_
4. size of outbreak at peak, *I*_max_ = max{*I*_*t*_, *t* ≥ 0}.

Since the simulated data contains times of onset of infectivity and host removal, the above quantities are indeed directly calculable. Additionally, the outbreak size trajectory, *I*_*t*_, over the course of an outbreak will be examined. Due to likely correlations between *m* and max (**t**^*R*^) − min (**t**^*I*^) and between *I*_max_ and *t*_peak_, these are plotted bivariately.

The time of outbreak peak, *t*_peak_, is interpolated between the first and last times that the outbreak size is within the range *cI*_max_ ≤ *I*_*t*_ ≤ *I*_max_, where 0 < *c* < 1, i.e.,


$$\begin{array}{c} t_{\text{peak}}=\frac{\text{max}\left\{t:I_{t}\geq cI_{\text{max}}\right\}-\text{min}\{t:I_{t}\geq cI_{\text{max}}\}}{2}. \end{array}$$ (10)

Calculating *t*_peak_ this way, rather than simply taking the time that the outbreak size reaches its maximum, avoids the complication of the outbreak hitting its maximum size more than once and, more importantly, aims to reduce the variance of its posterior-predictive distribution, and therefore produce a sharper test for model departure. Here *c* is chosen to be 0.3, since the GPPC outputs were not found to be sensitive to the particular value chosen.

## Supporting information

S1 Appendix Supplementary text. Detailed description of Metropolis-cooled MCMC routine, derivation of force of infection for Reed-Frost epidemic models, parameter estimation for WSSV SEIR-P model and diagnostic MCMC trace plots.
(PDF)
