# Supplementary material for: When and why direct transmission models can be used for environmentally persistent pathogens
Source: PLoS Comput Biol. 2021 Dec 1;17(12):e1009652. doi: 10.1371/journal.pcbi.1009652 (PMC8668103; doi:10.1371/journal.pcbi.1009652)
Supplement: S1 Appendix — Detailed description of Metropolis-cooled MCMC routine, derivation of force of infection for Reed-Frost epidemic models, parameter estimation for WSSV SEIR-P model and diagnostic MCMC trace plots. (PDF) [file pcbi.1009652.s001.pdf]

A Metropolis-cooled MCMC routine

A.1 SEIR likelihood

As stated in Materials and methods, the likelihood for the SEIR model with exponentially-distributed exposed and infectious lifetimes is

$$p(\,\mathbf{t}^E, \mathbf{t}^I, \mathbf{t}^R \,|\, \beta, \gamma, \delta\,) \propto \beta^{m-1} B(\mathbf{t}^E, \mathbf{t}^I, \mathbf{t}^R) \times \exp\Big\{ -\beta A(\mathbf{t}^E, \mathbf{t}^I, \mathbf{t}^R) \Big\} \times \cdots$$
$$\cdots \times \delta^m e^{-\delta \sum_{j=1}^m (t_j^I - t_j^E)} \times \gamma^m e^{-\gamma \sum_{j=1}^m (t_j^R - t_j^I)}.$$

where m is the final outbreak size (i.e. number of exposure, onset of infectivity and removal events) and t_κ^E (below) is the unobserved time of the index exposure

$$A(\mathbf{t}^E, \mathbf{t}^I, \mathbf{t}^R) = \int_{t_\kappa^E}^{\infty} S_t I_t \, dt$$
$$B(\mathbf{t}^E, \mathbf{t}^I, \mathbf{t}^R) = \prod_{i \neq \kappa} \Big\{ I_{t_i^E -} \Big\}$$

where $I_{t_i^E -}$ is the number of infectious hosts *immediately before* the ith exposure time.

The integral $\int_{t_\kappa^E}^{\infty} S_t I_t \, dt$ has an easily computable form, found in [1]

$$A(\mathbf{t}^E, \mathbf{t}^I, \mathbf{t}^R) = \sum_{j=1}^{m} \sum_{i=1}^{N} \big\{ \min(t_j^R, t_i^I) - \min(t_j^I, t_i^I) \big\}$$

where j sums over all exposure events and i sums over the entire host population. This quantity represents the total susceptible-infectious host contact time throughout the outbreak.

To explain the form of the likelihood, each exposed host, j, contributes terms $\delta e^{-(I_j - E_j)}$ and $\gamma e^{-(R_j - I_j)}$, since they spend Exp(δ) and Exp(γ) times in the E and I states, respectively. After exposure, all onsets of infectiousness and recoveries are observed. Ordering the exposure events, $t_\kappa^E = t_1^E < \cdots < t_m^E$, each exposure except the first also contributes a term

$$\beta S_{t_i^E -} I_{t_i^E -} \exp\Big\{ -\beta \int_{t_{i-1}^E}^{t_i^E} S_t I_t \, dt \Big\} \qquad i > 1$$

since the ith exposure event has hazard $\beta S_t I_t$ beginning at time E_{i-1}. Finally, there is the factor

$$\exp\Big\{ -\beta \int_{t_i^E}^{\max(\mathbf{t}^R)} S_t I_t \, dt \Big\}$$

representing the probability that no further exposure events occur. Since $S_{t_1^E -} S_{t_2^E -} \ldots S_{t_m^E -} = (N-1)(N-2)\ldots(N-m+1) =$ constant, these factors are omitted from the likelihood.

A.2 Markov chain Monte Carlo (MCMC)

Since we can write down the posterior density for γ, and within the MCMC routine described in the Materials and methods, samples of δ and $\mathbf{t}^E$ do not depend on previous samples of β, the main routine need only consist in sampling δ and $\mathbf{t}^E$ and recording these values along with A, B and t_κ^E. The parameter β can then be sampled from its full conditional distribution, which given an exponential prior $p(\beta) \sim \text{Exp}(\omega_\beta)$ is

$$p(\beta|\delta, \gamma, \mathbf{t}^E, \mathbf{t}^I, \mathbf{t}^R) \sim \Gamma(m, A + \omega_\beta)$$

Since the full posterior distribution of parameters and exposure times, $p(\,\beta, \delta, \gamma, \mathbf{t}^E \,|\, \mathbf{t}^I, \mathbf{t}^R\,)$ is intractable, we use MCMC methods to sample dependent sequences $\beta_i, \delta_i, \gamma_i, \mathbf{t}_i^E$ as follows:

We first set initial values for the parameters and exposure times, $\beta_0, \delta_0, \gamma_0, \mathbf{t}_0^E$ and then, for some $T \geq 1.0$ (the *temperature* - see below), iterating through the following steps for $i = 1, \ldots, n$:

1. Update δ via Metropolis-Hastings, i.e., propose $\delta' \sim N(\delta^{i-1}, \sigma_\delta^2)$ and with probability a_1 set $\delta^i = \delta'$, otherwise $\delta^i = \delta^{i-1}$, where

$$a_1 = \min\left(1, \frac{f_T(\delta', \mathbf{t}^E|\mathbf{t}^I, \mathbf{t}^R)}{f_T(\delta^{i-1}, \mathbf{t}^E|\mathbf{t}^I, \mathbf{t}^R)}\right)$$

   f_T is the marginal conditional density for $\delta, \mathbf{t}^E$ at temperature T (see below). The parameter σ_δ is tuned during an number of iterations in order to get an acceptance rate of between 20% and 40%.

2. Choose an exposure time to update (with index j) uniformly at random. Propose $\mathbf{t}^{E'}$, where $t_k^{E'} = t_{k,i-1}^E$ for $k \neq j$ and $t_j^I - t_j^{E'} \sim \text{Exp}(\delta^i)$ and with probability a_2 set $t_{j,i}^E = t_j^{E'}$, otherwise set $t_{j,i}^E = t_{j,(i-1)}^E$ (all other exposure times are unaltered), where

$$a_2 = \min\left(1, \frac{f_T(\delta^i, \mathbf{t}^{E'}|\mathbf{t}^I, \mathbf{t}^R)}{f_T(\delta^i, \mathbf{t}_{(i-1)}^E|\mathbf{t}^I, \mathbf{t}^R)} \exp\{-\delta^i(t_j^{E'} - t_{j,i}^E)\}\right)$$

3. Update β by the Gibbs sampler, i.e. sample from the full conditional distribution $\beta^i \sim \Gamma(m, A+\omega_\beta)$.

The above follows a fully-centred parameterisation, as discussed by Neal and Roberts for the SIR model [1]. Note that there is no need to sample γ as part of the above routine, since having assumed an exponentially-distributed prior $p(\gamma) = \omega_\gamma e^{-\omega_\gamma \gamma}$, its posterior density is

$$p(\gamma|\mathbf{t}^E, \mathbf{t}^I, \mathbf{t}^R) \propto \gamma^m e^{-\gamma \sum_{j=1}^m (t_j^R - t_j^I)} \times e^{-\omega_\gamma \gamma}$$

and therefore

$$p(\gamma|\mathbf{t}^E, \mathbf{t}^I, \mathbf{t}^R) \sim \text{Gamma}(m+1, \sum_{j=1}^m (t_j^R - t_j^I) + \omega_\gamma)$$

Due to the high dimensionality of the sample space and the likelihood function perhaps having local maxima, the sampling chains can sometimes be slow to converge to stationarity and even become stuck at certain parameter values, with an acceptance ratio going towards zero. *Metropolis coupled* MCMC, or $(\text{MC})^3$ [2] is the strategy adopted here to alleviate poor mixing and is summarised as follows: several of the above chains are run with several closely spaced temperatures $1.0 = T_1 < T_2 < \cdots < T_r$. The first chain, with temperature 1.0, is termed the *cold chain* and is the only chain from which we obtain samples. The other chains are known as the *heated* chains. After performing a fixed number of iterations for each chain in parallel, two are selected uniformly at random, with temperatures T' and T'' and current states $X' = \beta', \delta', \mathbf{t}^{E'}$ and $X'' = \beta'', \delta'', \mathbf{t}^{E''}$. The states are then exchanged with probability a_3, where

$$a_3 = \min\left(1, \frac{f_{T_1}(X''|\mathbf{t}^I, \mathbf{t}^R)}{f_{T_1}(X'|\mathbf{t}^I, \mathbf{t}^R)} \frac{f_{T_2}(X'|\mathbf{t}^I, \mathbf{t}^R)}{f_{T_2}(X''|\mathbf{t}^I, \mathbf{t}^R)}\right)$$

Although the samples from the heated chains are ultimately discarded, the method has the advantage that is easily parallelised on a multi-core machine. For this work, this was achieved using Python's multiprocessing module. Six chains were run in parallel with the temperatures $T = 1.00, 1.02, 1.04, 1.06, 1.08, 1.10$ and exchanges of state were attempted every 400 iterations.

We can therefore marginalise the posterior density at temperature T, obtaining f_T which is, for $p(\delta) \sim \mathrm{Exp}(\omega_\delta)$

$$
\begin{aligned}
f_T(\delta, \mathbf{t}^E | \mathbf{t}^I, \mathbf{t}^R) &\propto p(\,\delta, \mathbf{t}^E \,|\, \mathbf{t}^I, \mathbf{t}^R\,)^{\frac{1}{T}} \\
&\propto \left\{ \int\!\!\int p(\,\mathbf{t}^E, \mathbf{t}^I, \mathbf{t}^R \,|\, \beta, \delta, \gamma\,) p(\beta) p(\delta) p(\gamma) d\beta\, d\gamma \right\}^{\frac{1}{T}} \\
&\propto \left\{ \prod_{i \neq \kappa} \{I_{t_i^E}\} \, (A + \omega_\beta)^{-m} \times \delta^m \exp\Big\{ -\delta \Big(\sum_{j=1}^{m} (t_j^I - t_j^E) + \omega_\delta \Big) \Big\} \right\}^{\frac{1}{T}}
\end{aligned}
$$

and for $p(\delta) \sim \mathrm{U}(0, 10)$

$$
f_T(\delta, \mathbf{t}^E | \mathbf{t}^I, \mathbf{t}^R) \propto \left\{ \prod_{i \neq \kappa} \{I_{t_i^E}\} \, (A + \omega_\beta)^{-m} \times \delta^m \exp\Big\{ -\delta \sum_{j=1}^{m} (t_j^I - t_j^E) \Big\} \right\}^{\frac{1}{T}} \times \mathbf{1}_{\delta \in (0,10)}.
$$

B Force of infection in Reed-Frost epidemic model

Lotz and Soto in [3] adopt a Reed-Frost model in order to describe transmission of WSD among shrimp in a controlled experiment in which transmission may either be exclusively direct, via ingestion of dead infected shrimp, or exclusively environmental, via cohabitation with a live, infected shrimp. Reed-Frost (see e.g., [4]) is a discrete time model of the numbers of susceptible (S_t), infected (I_t) and removed (R_t) individuals at each of a series of closely spaced time points, separated by a duration Δt. The transmission parameter β is the probability of transmission from some particular infected individual to some other particular susceptible individual during one time step, so that the probability of some susceptible individual not being infected during one time step is

$$(1 - \beta)^{I_t} \tag{1}$$

and the probability that there is transmission from *at least one* infected individual to this specified susceptible is therefore

$$1 - (1 - \beta)^{I_t} \tag{2}$$

and the expected number of new infections occuring is

$$S_t(1 - (1 - \beta)^{I_t}). \tag{3}$$

The force of infection is therefore

$$\frac{1 - (1 - \beta)^{I_t}}{\Delta t} \approx \frac{\beta I_t}{\Delta t}. \tag{4}$$

C Estimation of α, ϵ and ρ for SEIR-P model of WSD in shrimp

C.1 Estimation of pathogen decay rate, ρ

By a challenge experiment in which *P. monodon* were immersed in sterile seawater that had been spiked with WSSV a variable number of days prior to immersion, Kumar et.al. were able to estimate how long a known quantity of WSSV remains viable in seawater under laboratory conditions. Ten experimental and one control bucket were filled with 10l of sterile seawater. To the experimental buckets pure WSSV

was added to a final concentration of 1000 virion ml^{-1}, meaning that around 10^7 particles were present in each bucket. On days 0 up to 18, 10 juvenile *P. monodon* were added to one of the unoccupied buckets and all shrimp were monitored at 8h intervals for mortality or signs of WSSV infection. Dead shrimp were removed from the buckets and proportions of living shrimp were recorded daily for each bucket. The authors found that under the conditions of the experiment seawater-borne WSSV remains infective for up to 12 days.

The plots given in [5, Figure 2] for buckets 0 to 8 indicate similar rates of mortality across these buckets, suggesting that the WSSV lost little of its infectivity during the first eight days in seawater. Total mortality occurred at around the four day mark following introduction to the 0 to 8-day buckets. A reduction in mortality rates is then noticeable following immersion in the 10 day and 12 day buckets, where 100% mortality was observed each at the 7 day mark (i.e 7 days after immersing the shrimp). No mortalities occurred following immersion in the 14, 16 and 18 day buckets, at least during the experimental period, suggesting that the amount of viable WSSV had decayed significantly by 12-14 days in seawater. Since at 8 days there was still a sufficient quantity of WSSV to inoculate all 10 of the shrimp, we expect that the mean infectious lifetime a WSSV particle in seawater to be no less than 8 days, giving pathogen decay rate, ρ, no greater than 0.005 h^{-1}.

C.2 Estimation of environmental transmission rate, α

The second column of Table A contains the time in hours, t_{100}, from immersion to 100% mortality of shrimp in each of the buckets, labelled by the number of days after the introduction of WSSV to the bucket that the shrimp were immersed. A lower, order of magnitude, estimate for α (indirect rate of WSD transmission) can be obtained from the same data by assuming that mortality comes immediately upon infection (thus underestimating the infectivity) and assuming that the pathogen density remained at its initial level of 1000 virion ml^{-1}, at least for buckets 0 to 8, where the rates of mortality were similar.

The transmission rate α should be no more than $10/(10^3 \times t_{100})$, since 10 exposures occurred in t_{100} hours, and the rate of new exposures is $\alpha P = 10^3\alpha$. These values are in Column 3 of Table A. Similarly, upper estimates for each bucket by supposing that 100% of the exposures had happened at t_{100} - 48, i.e. fixing a period of 2 days between exposure and death for each shrimp since no deaths occurred before 2 days, post-immersion. These are in Column 4. Taking medians, we can say that α is of the order of 10^{-4} ml virion^{-1} h^{-1}.

Bucket	t_{100} h	α ml virion^{-1} h^{-1}	
0	96	1.0×10^{-04}	2.1×10^{-4}
2	120	8.3×10^{-05}	1.4×10^{-4}
4	96	1.0×10^{-04}	2.1×10^{-4}
6	72	1.4×10^{-04}	4.2×10^{-4}
8	96	1.0×10^{-04}	2.1×10^{-4}

Table A: **From graphical plots by Kumar, et.al.** ([5, Fig 2]). Buckets are labelled by time in days between addition of WSSV to bucket and immersion of shrimp. t_{100} - the time in hours to 100% mortality. Upper and lower estimates of α are given to 1 decimal place and calculations are described in main body of text.

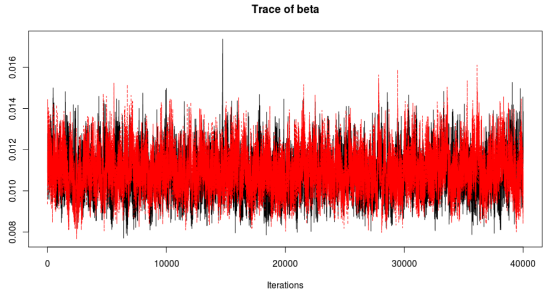

(a) Long-lived

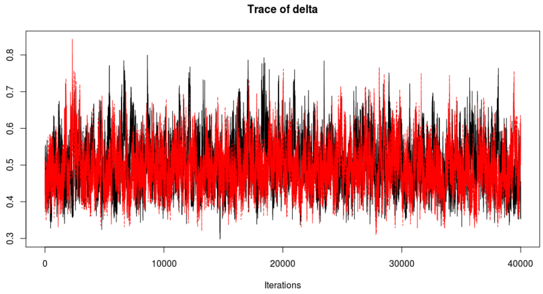

(b) Long-lived

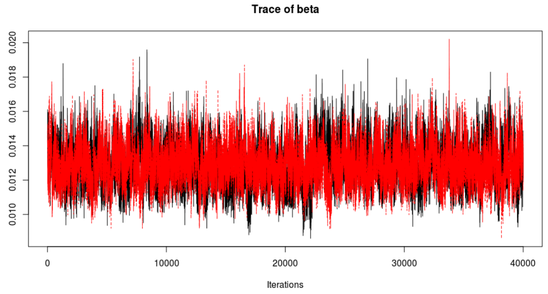

(c) Intermediate

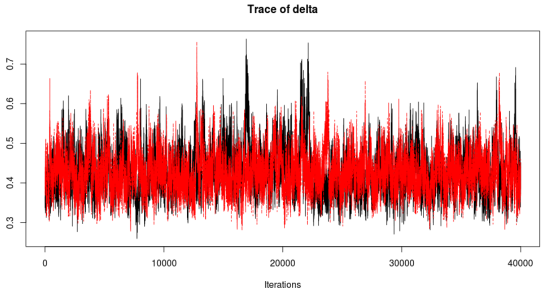

(d) Intermediate

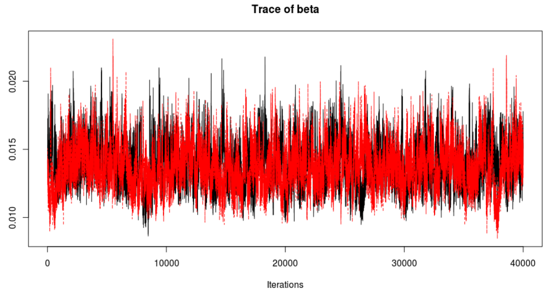

(e) Short-lived

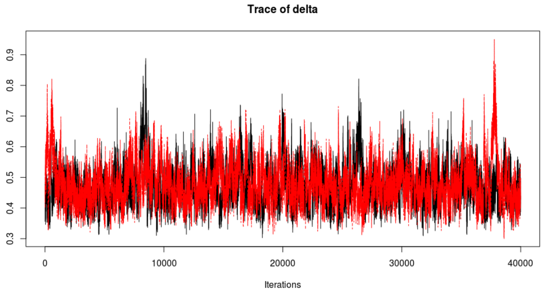

(f) Short-lived

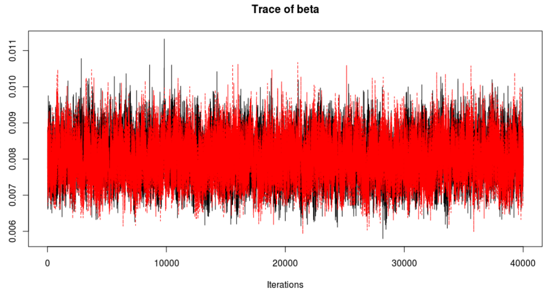

(g) Direct transmission only

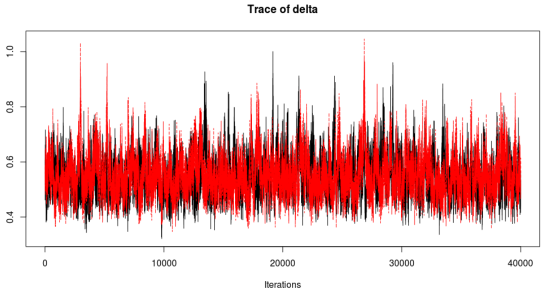

(h) Direct transmission only

Figure A: Trace plots using R `coda` package. In the case of *long-lived pathogen* (b), a restricted prior was adopted for δ: $p(\delta) \sim \mathrm{U}(0, 10.0)$ in order to aid mixing. In all other cases $p(\beta), p(\delta) \sim \exp(0.001)$ independently. Trace plots show all iterations, including those later discarded for burn in, in order to demonstrate convergence of two independent chains to stationarity.

References

[1] Neal P, Roberts G. A case study in non-centering for data augmentation: Stochastic epidemics. Statistics and Computing. 2005;15(4):315–327. doi:10.1007/s11222-005-4074-7.

[2] Altekar G, Dwarkadas S, Huelsenbeck JP, Ronquist F. Parallel Metropolis coupled Markov chain Monte Carlo for Bayesian phylogenetic inference. Bioinformatics. 2004;20(3):407–415. doi:10.1093/bioinformatics/btg427.

[3] Soto MA, Lotz JM. Epidemiological Parameters of White Spot Syndrome Virus Infections in Litopenaeus vannamei and L. setiferus. Journal of Invertebrate Pathology. 2001;78(1):9–15. doi:10.1006/jipa.2001.5035.

[4] Abbey H. An Examination of the Reed-Frost Theory of Epidemics. Human Biology. 1952;24(3):201.

[5] Satheesh Kumar S, Ananda Bharathi R, Rajan JJS, Alavandi SV, Poornima M, Balasubramanian CP, et al. Viability of white spot syndrome virus (WSSV) in sediment during sun-drying (drainable pond) and under non-drainable pond conditions indicated by infectivity to shrimp. Aquaculture. 2013;402-403:119–126. doi:10.1016/j.aquaculture.2013.04.001.
